# Phase Engineering of WO_3_ Nanoparticles Towards Efficient Photochromic Nanocomposites

**DOI:** 10.3390/molecules31193460

**Published:** 2026-09-28

**Authors:** Nicola Caggiano, Carlo Nazareno Dibenedetto, Elisabetta Fanizza, Pierluigi Lasala, Annamaria Panniello, Teresa Sibillano, Cinzia Giannini, Maria Lucia Curri, Marinella Striccoli

**Affiliations:** 1 Dipartimento di Chimica, Università degli Studi di Bari, Via Orabona 4, 70126 Bari, Italy; nicola.caggiano@uniba.it (N.C.); elisabetta.fanizza@uniba.it (E.F.); perluigi.lasala@uniba.it (P.L.); 2 CNR-Istituto per i Processi Chimico Fisici (CNR-IPCF), Via Orabona 4, 70126 Bari, Italy; annamaria.panniello@cnr.it (A.P.); marialucia.curri@uniba.it (M.L.C.); 3 CNR-Istituto di Cristallografia (CNR-IC), Via Amendola, 122/O, 70126 Bari, Italy; teresa.sibillano@cnr.it (T.S.); cinzia.giannini@cnr.it (C.G.)

**Keywords:** tungsten oxide, photochromism, sol–gel synthesis, nanocomposites, poly(vinylpyrrolidone)

## Abstract

Tungsten oxide (WO_3_) is an attractive phase-sensitive chromogenic material whose photoinduced optical response produces reversible coloration of interest light responsive optical devices. Here, WO_3_ nanoparticles (NPs) are synthesized via a simple surfactant-free sol–gel precipitation route by systematically varying acid content and reaction temperature then characterized to establish a correlation among synthesis conditions, crystalline phase morphology and photochromic response. Morphological, structural and optical characterizations show that by gradually heating the precursors at 90 °C and varying the acid content, predominantly sheet-like orthorhombic and hydrated-orthorhombic WO_3_ NPs are yielded, characterized by an intense yellow color, optical band-gap values of approximately 2.4 eV, and negligible photochromism. In contrast, in a highly acidic environment. the temperature tuning promotes the formation of rod-like WO_3_ NPs with whitish color and higher band gap. The crystalline structures change from hexagonal to hexagonal hydrogen bronze phase, decreasing the reaction temperature. Under UV irradiation, these samples exhibit markedly higher photochromic efficiency than the orthorhombic phase, up to 18-fold higher, due to more accessible proton intercalation pathways offered by the hexagonal tunnel structure. The incorporation into poly(vinylpyrrolidone) allows the production of nanocomposite films that preserve and enhance the photochromic response. Overall, the reaction temperature is identified as the key synthetic parameter controlling WO_3_ crystalline phase that consequently affects photochromic behaviors, while polymer integration provides a practical route toward processable, flexible, and device-compatible photochromic coatings.

## 1. Introduction

Tungsten oxide (WO_3_) is a structurally versatile transition metal oxide whose optical properties are highly sensitive to crystal symmetry, hydration state, defect chemistry, and ion transport pathways [1]. This sensitivity triggers the well-established chromogenic behavior, namely the reversible modulation of optical absorption in response to electrical, chemical, or optical stimuli [2]. Among these phenomena, photochromism is particularly attractive because it enables light-induced coloration without the need for an externally applied electrical bias. This light-driven switching mechanism makes WO_3_ a promising functional material for a wide range of smart and adaptive technologies. Because the process can be initiated and controlled using light, WO_3_-based systems offer the potential for low-power or self-activated optical modulation, eliminating the need for continuous electrical input during the coloration process. This feature is especially advantageous for applications such as smart windows, where the transmission of visible and near-infrared light can be dynamically regulated [3], and optical and chemical sensors, where light-induced coloration can serve as an indicator of environmental or chemical changes [4]. Furthermore, the combination of photochromism with the well-known electrochromic, photocatalytic [5], and sensing properties of WO_3_ makes it a versatile material for developing multifunctional and light-responsive devices [6,7]. Consequently, understanding and controlling the photochromic response of WO_3_ is of significant interest for the development of next-generation adaptive optical systems, energy-efficient technologies, and environmentally responsive functional materials. In the literature, the photochromic response of WO_3_ is generally associated with the generation of electron–hole pairs under irradiation, followed by surface-mediated reactions involving adsorbed water and the insertion of charge-compensating protonic species into the oxide lattice [8,9]. The following reactions provide a simplified description of the reactivity of WO_3_ under UV irradiation.

$${\mathrm{WO}}_{3}+h\nu \to {\mathrm{WO}}_{3}^{*}+e^{-}+h^{+}$$

$$h^{+}+\frac{1}{2}H_{2}O\to \frac{1}{4}O_{2}+H^{+}$$

$$xH^{+}+xe^{-}+WO_{3}\to H_{x}W_{x}^{5+}W_{1-x}^{6+}O_{3}$$

The resulting coexistence of W^5+^ and W^6+^ centers give rise to intervalence charge transfer (IVCT), which produces the characteristic blue coloration of reduced tungsten oxide [8,9]. Although this mechanism is often described in terms of proton insertion, the efficiency and reversibility of the process depend on several coupled factors, including carrier generation and separation, surface hydration, density of oxygen vacancies, accessibility of interstitial sites, and the structural pathways available for ion transport [2,8,9,10,11].

Crystal structure therefore plays a central role in determining WO_3_ photochromism. WO_3_ can exist in several polymorphs, including monoclinic, orthorhombic, tetragonal, hexagonal, and hydrated and hydrogenated derivatives, each characterized by a distinct connectivity and distortion of the WO_6_ octahedral framework [12,13]. These structural differences directly influence electronic states, lattice free volume, ion insertion energetics, and diffusion kinetics. In particular, hexagonal WO_3_ contains one-dimensional tunnels formed by corner-sharing WO_6_ octahedra, which can provide more accessible pathways for the insertion and migration of small cations than the denser orthorhombic or monoclinic structures [14,15]. Hydration and hydrogenation can further modify these pathways by expanding the lattice, altering hydrogen-bonding networks, and facilitating proton transfer. Conversely, compact phases or strongly distorted frameworks may impose higher kinetic barriers to ion insertion and thereby limit the photochromic response.

The structural sensitivity of WO_3_ becomes even more pronounced at the nanoscale [12,13]. Reducing the material to nanoscale shortens solid-state diffusion distances, increases the surface area and the abundancy of interfacial sites, and enhances the influence of surface hydration and defect states [16,17,18,19,20]. At the same time, the high surface energy of nanostructured WO_3_ makes phase stability strongly dependent on synthesis conditions. Wet chemical precipitation and sol–gel routes are particularly attractive in this context because they offer low-temperature scalable processing and allow systematic control over precursor speciation, acidity, temperature and dehydration kinetics [21,22]. Relatively small changes in acidity, temperature, heating profile and precursor concentration can alter nucleation and condensation kinetics, promoting anisotropic growth, and shifting the balance among anhydrous and hydrated polymorphs [23]. Abbaspoor et al. demonstrated that solution pH affects WO_3_ particle size, optical bandgap and phase evolution in HCl-acidified sodium tungstate systems, while calcination promotes conversion toward monoclinic WO_3_ [24]. Marques et al. further demonstrated that careful control of acidity and the use of structure-directing agents enable the synthesis of WO_3_ nanoparticles (NPs) with either phase-pure or mixed-phase compositions [25].

These studies highlight the sensitivity of WO_3_ formation to the chemical environment, but they do not fully distinguish the respective roles of acidification and reaction temperature in determining phase composition and photochromic performance under otherwise comparable conditions. This distinction is important because acidification and temperature affect different stages of particle formation. Acidification controls tungstate protonation, condensation, and precursor speciation, whereas temperature influences nucleation, crystal growth, dehydration, and polymorph stabilization. Moreover, the way in which the target temperature is reached may be as relevant as the nominal reaction temperature itself, since gradual and rapid heating can generate different supersaturation profiles and growth regimes. A systematic comparison of these parameters within the same synthetic framework is therefore required to identify the conditions that govern WO_3_ phase selection and to relate the resulting structures to their photochromic response. Consequently, synthesis is not merely a means of controlling particle size or morphology, but a primary tool for regulating phase composition and, ultimately, photochromic behavior. In addition to controlling phase formation and photochromic response, practical implementation requires the integration of WO_3_ nanoparticles into processable solid-state architectures.

Bare nanoparticle powders or particulate coatings may exhibit limited adhesion, mechanical stability, and film uniformity, restricting their direct use in optical devices. Embedding WO_3_ nanoparticles in an optical transparent polymer matrix provides a straightforward strategy to combine the optical activity of the inorganic component with the film-forming and mechanical properties of the organic host [26]. The selected polymer needs to also be chemically compatible with the nanoparticle surface and sufficiently permeable to water and oxygen so as not to hinder the interfacial processes involved in photochromism.

Poly(vinylpyrrolidone) (PVP) was demonstrated to be a suitable host matrix because of its optical transparency, processability, polar functionality, and affinity for metal oxide surfaces [27,28,29,30]. Spectroscopic investigations have shown that the carbonyl groups of PVP can interact with WO_3_, contributing to nanoparticle dispersion and stabilization within the polymer matrix [27,28]. PVP may also influence the local hydration environment and the stability of photoinduced reduced tungsten species, although its specific contribution to proton transfer and redox processes depends on composition and processing conditions [29]. Previous studies further indicate that PVP can support the reversibility of the coloration–bleaching cycle. Its transparency in the visible region minimizes its direct contribution to the measured optical response, while its film-forming properties make it a practical platform for evaluating WO_3_ in a processable coating configuration [30].

Here, we performed a systematic investigation of synthesis conditions, investigating the correlation between preparatory parameters, such as the acid amount added to the precursors and the heating processes, nanoparticle shape, crystalline phase composition, and photochromic response. WO_3_ NPs were synthesized via a simple surfactant-free, acid-catalyzed, sol–gel precipitation route from sodium tungstate dihydrate acidified with HCl, without structure-directing additives or post-synthetic treatments. Acidification and thermal conditions were systematically varied to investigate their effects on morphology, phase composition, optical properties, and photochromic behavior. The resulting materials were characterized by transmission electron microscopy (TEM), quantitative X-ray powder diffraction (XRPD), and diffuse reflectance spectroscopy (DRS).

The most suitable WO_3_ NPs, in terms of photochromic response and reaction yield, were then incorporated into PVP films at different NP loadings and tested under 254 and 365 nm irradiation. Overall, the study establishes a synthesis–structure–photochromism relationship and evaluates the transfer of the NP response to processable WO_3_/PVP coatings.

## 2. Results and Discussion

WO_3_ NPs were prepared through an acid-induced sol–gel precipitation route, and the resulting materials were investigated to determine how the degree of acidification and the thermal conditions influence particle morphology, crystalline phase, optical properties, and photochromic behavior. The two synthetic series, WO_3_A and WO_3_T described in Section 3, were therefore compared to distinguish the influence of acid addition from that of reaction temperature and heating profile on the photochromic behavior.

### 2.1. Effect of Acidification

The first series (WO_3_A) was prepared by gradual heating up to 90 °C by progressively increasing the amount of HCl added to the sodium tungstate solution.

Upon acid addition, tungstate ions are protonated to form tungstic acid intermediates, as indicated by the development of a pale-yellow color in the reaction mixture. Tungstic acid subsequently undergoes condensation and dehydration, leading to the formation of WO_3_ according to the simplified reactions [24]:

Na_2_WO_4_∙2H_2_O + 2HCl → H_2_WO_4_ + 2NaCl + 2H_2_O

H_2_WO_4_ → WO_3_ + H_2_O

Yields of the reaction were determined as follows:

$$\mathrm{Yield}\;\left(\%\right)=\frac{{{\mathrm{WO}}_{3}}_{\mathrm{Obt}}}{{{\mathrm{WO}}_{3}}_{\mathrm{th}}}\cdot 100$$

where ${{\mathrm{WO}}_{3}}_{\mathrm{Obt}}$ is the mass of the dried product recovered after purification of the synthesized WO_3_, and ${{\mathrm{WO}}_{3}}_{\mathrm{Th}}$ is the theoretically expected mass of 2.32 g (0.01 mol).

The product yields ranged from 64 to 72%, indicating that increasing the amount of HCl within the investigated interval did not substantially affect the overall recovery of the tungsten oxide product. TEM analysis shows that WO_3_A1.7, WO_3_A2.1 and WO_3_A2.5 consisted predominantly of aggregated sheet-like structures, with no evident systematic variation in morphology across the first three acidification conditions (Figure 1a–c). Only for the sample prepared with the highest HCl amount, WO_3_A2.9, a second anisotropic population emerged, with rod-like shapes together with the prevailing sheet-like structures (Figure 1d). This morphological change suggests that the stronger acidification modified the relative rate of tungstate species protonation, condensation, and crystal growth, supporting anisotropic development [24,31]. Nevertheless the persistence of sheet-like structures throughout the series indicates that increasing acid addition alone does not induce a complete transition to a different growth regime. The optical properties were similarly weakly affected by the degree of acidification. 

The optical band gap values, estimated from Tauc plots assuming an indirect allowed transition, yielded E_g_ values between 2.41 ± 0.02 eV and 2.44 ± 0.02 eV (Figure 1e), consistent with their intense yellow color and appreciable absorption in the visible region. The absence of a systematic shift in E_g_ indicates that the modest morphological and structural changes induced by increasing HCl addition did not substantially modify the electronic structure of the dominant phase. Structural characterization was carried out by crystallographic measurements. X-ray diffraction patterns of WO_3_A1.7 and WO_3_A2.9 show profiles well fitted with a hydrated orthorhombic and a pure orthorhombic phase, respectively (Figure 1f,g, Rietveld refinements in Figures S1 and S2 in the Supplementary Materials). The patterns of WO_3_A2.1 and WO_3_A2.5 (Figure S3, Rietveld refinements in Figures S4 and S5) show the crystallographic phases of the previous samples, respectively. In addition, two more synthesis were carried out at the highest HCl amount, with final ramp temperatures of 75 °C and 60 °C (samples named WO_3_A2.9R_75 and WO_3_A2.9R_60, respectively), to investigate the effect of temperature on the crystallographic features of the WO_3_ NPs. The results, reported in Figures S6–S8 and summarized in Table S2 of the Supplementary Materials, reveal that, in gradual heating processes, a decrease in temperature induces the formation of mixed crystalline phases, introducing hexagonal and hexagonal hydrogen bronze structures.

### 2.2. Effect of Reaction Temperature

To isolate the effect of heating conditions, a second series of experiments (WO_3_T) was performed using a fixed HCl addition of 2.9 mL while varying the reaction temperature from 45 °C to 90 °C. Unlike the acidification series, these experiments employed a direct immersion of the reactor into a preheated oil bath. The resulting rapid thermal input may alter the nucleation and growth of NPs.

After synthesis and drying, the samples of the WO_3_T series prepared at reaction temperatures of 90 °C, 75 °C, or 60 °C appeared predominantly white compared to the yellow color of WO_3_A samples. TEM micrographs of WO_3_T90, WO_3_T75 and WO_3_T60 show aggregated NPs with a well-defined rod-like shape and typical lengths of approximately 100 nm (Figure 2a–c). Statistical analysis of the size dispersion of the samples, reported in Figure S14, allows us to estimate the average dimension of the nanorod structures, with lengths of several dozens of nm and widths around 20–50 nm, with average aspect ratios ranging from 3.3 to 5.5 (see Figure S14 in the SI). However, the size dispersion was very high, approaching values of 30–40%.

A distinct behavior was observed for the sample synthesized at 45 °C. After purification and drying, WO_3_T45 displayed two visually distinguishable components, one bluish and one yellow. As described in the Section 3, low-speed centrifugation enabled their separation into a bluish fraction, denoted WO_3_T45B, and a yellow fraction, denoted WO_3_T45Y. TEM analysis showed that the two fractions also differed morphologically (Figure 2d,e), supporting the presence of distinct particle populations. The comparison between WO_3_T90 and WO_3_A2.9 is particularly informative, since both samples were prepared using the same amount of HCl and the same nominal reaction temperature, while differing primarily in the heating protocol. WO_3_A2.9, obtained under gradual heating from room temperature, consisted predominantly of sheet-like structures with a minor rod-like contribution. By contrast, WO_3_T90, prepared by direct immersion of the reactor into a preheated oil bath, was dominated by rod-like nanoparticles. This comparison indicates that the overall heating conditions of the synthesis, rather than the nominal reaction temperature alone, plays a decisive role in directing particle growth.

Gradual heating allows a progressive evolution of precursor protonation, condensation, and structural rearrangement, supporting the coexistence of sheet-like and rod-like morphologies. In contrast, rapid heating modifies the supersaturation and nucleation profiles and promotes anisotropic crystal growth, leading to the formation of nanorods. Although the specific growth mechanism was not directly investigated, the observed morphological differences identify the heating profile as an important kinetic parameter in the formation of WO_3_ nanoparticles.

The thermal conditions also markedly affected the optical properties of the WO_3_ nanoparticles. Tauc analysis of the Kubelka–Munk-transformed diffuse reflectance spectra yielded optical band-gap values between 3.15 eV and 3.29 eV for WO_3_T90, WO_3_T75, WO_3_T60, and WO_3_T45B (Figure 2f and Table 1). These values are substantially higher than the value of about 2.4 eV measured for the orthorhombic-rich samples of the acidification series. The wider band gaps are consistent with the white appearance of these powders and their lower absorption in the visible region.

The two fractions isolated from WO_3_T45 displayed markedly different optical characteristics. The bluish WO_3_T45B fraction exhibited an E_g_ value of 3.15 eV, comparable to the other samples of the same series, whereas the yellow WO_3_T45Y fraction showed a substantially lower E_g_ of 2.55 eV. These differences indicate that the two fractions are not only morphologically distinct but also possess different optical and electronic characteristics, consistent with the different crystalline phase compositions established by XRPD analysis.

XRPD analysis provides direct evidence that the heating conditions during the synthetic process governed the phase composition of the WO_3_ NPs (Figure 3). Quantitative phase fractions obtained by Rietveld refinements, together with the reaction yields and optical band gaps of each sample, are summarized in Table 1 and Table S3. The experimental XRPD patterns are consistent with the reference reflections of the identified crystalline phases (Figure 3), supporting the qualitative phase assignments. The samples synthesized at 90 °C and 60 °C contain two crystalline phases. WO_3_T90 consists predominantly of hexagonal WO_3_, together with a 24.3% contribution of orthorhombic structure, whereas WO_3_T60 contains mainly hexagonal WO_3_, with a minor (12.9%) fraction of hexagonal hydrogen bronze structure (WO_3_H_0.07_). By contrast, WO_3_T75 diffraction pattern is assigned to a pure hexagonal phase, within the accuracy of the refinement. For WO_3_T90 and WO_3_T60, the coexistence of the two crystalline phases is not accompanied by the presence of clearly distinguishable particle populations, as previously pointed out by TEM micrographs, showing morphologically homogeneous ensembles of rod-like nanostructures (Figure 2a–c). This suggests that, at 60 °C and 90 °C, the two phases are associated with particle populations of similar morphology rather than with macroscopically distinct fractions. Moreover, the TEM measurements do not allow for resolving the spatial distribution of the phases within individual nanostructures.

The sample crystalline phase, as shown in Figure 3 and Table 1, can be related to temperature reaction conditions. At 90 °C, even though the hexagonal structure is predominant, the higher temperature supports partial evolution toward the orthorhombic polymorph, in agreement with the reported tendency of higher synthesis temperatures to stabilize denser WO_3_ phases in hydrothermal systems [32]. At 75 °C, a pure hexagonal phase was obtained under the applied reaction conditions, suggesting that this temperature provides a favorable balance among nucleation, crystal growth, and dehydration. At 60 °C, the material remained predominantly hexagonal but contained a minor hexagonal hydrogen bronze contribution, indicating that the lower thermal input increasingly limited dehydrogenation during crystallization. Replicated synthesis of the samples WO_3_T75 and WO_3_T60, carried out to check the reproducibility of the structural and optical features, confirms the presence of the same crystallographic phases and demonstrates reliable photochromatic efficiency.

The effects of thermal conditions at the final temperatures of 75 °C and 60 °C during the synthesis (fast and gradual heating approach), are also compared. The results, resumed in Tables S2 and S3 of the SM, indicate that the gradual heating up to 75 °C induces the formation of mixed crystallographic phases (hexagonal and orthorhombic), in place of the pure hexagonal one formed by direct fast heating at the same temperature. Similarly, a mix of three phases (hexagonal, hexagonal hydrogen bronze and hydrated orthorhombic) resulted from the gradual heating at 60 °C, compared with the hexagonal and hexagonal hydrogen bronze phases obtained by direct heating. The experimental findings clearly indicate that rapid heating limits the development of orthorhombic phase promoting hexagonal or hydrogenated structures. Decreasing the temperature to 45 °C, a different behavior was observed. WO_3_T45B consisted exclusively of hexagonal hydrogen bronze nanoparticles, whereas WO_3_T45Y contained both hexagonal hydrogen bronze and hydrated orthorhombic phases, in proportions of 57.1% and 42.9%, respectively.

Taken together, these results indicate that lowering the reaction temperature promoted the stabilization of hydrogenated WO_3_ polymorphs, most likely because of the reduced extent of dehydration during crystallization [33]. Conversely, the intermediate temperature of 75 °C supported pure hexagonal phase, while increasing the temperature to 90 °C led to the appearance of an orthorhombic contribution. The effects of structural and optical differences between the WO_3_T and WO_3_A series on the photochromic response are described and discussed in the following section.

### 2.3. Phase-Dependent Photochromic Behavior of WO_3_ NPs

The photochromic response of the WO_3_ NPs was evaluated on solid-state films, prepared as described above and irradiated with an UV lamp at 254 nm. The samples of the WO_3_A series showed only a negligible photochromic response. Despite prolonged irradiation for up to 80 min, their diffuse reflectance spectra remained essentially unchanged (Figure S14), with calculated ΔO.D._450nm_ values of approximately 0.008. This very low photochromic response is consistent with the predominance of orthorhombic and hydrated orthorhombic WO_3_ phases, which provide a less favorable structural environment for the photochromic process [14,15]. A markedly different behavior was observed for the samples belonging to the WO_3_T series, for which the influence of crystalline phase composition became evident. All samples containing hexagonal or hexagonal hydrogen bronze WO_3_ phases underwent a visible color change within the first 5 min of UV exposure, followed by a progressive modification of the differential optical density that approached a plateau after approximately 25 min. The diffuse reflectance spectra of WO_3_T75 at increasing irradiation times are shown in Figure 4a, while the corresponding data for the other samples of the same series are reported in Figure S15. WO_3_T90 exhibited a moderate photochromic response, with ΔO.D._450nm_ = 0.08, accompanied by a color change from white to pale blue (Figure 4b), while pure hexagonal phase WO_3_T75 reached ΔO.D._450nm_ = 0.15.

Both materials contained hexagonal WO_3_, but WO_3_T90 also included a 24.3% orthorhombic contribution and displayed an approximately 47% lower ΔO.D._450nm_ than WO_3_T75. This decrease confirms the detrimental effect of the orthorhombic fraction on the overall optical response. In agreement, the response of WO_3_T75 was almost 19 times higher than that of the orthorhombic-rich samples from the acidification series. This substantial difference supports the central role of the hexagonal phase in enabling photochromic coloration.The strongest photochromic responses were recorded for WO_3_T60 and WO_3_T45B, which reached ΔO.D._450nm_ values of 0.18 and 0.20, respectively. Both samples contain hexagonal hydrogen bronze WO_3_, suggesting that structural hydrogen may further favor the coloration process. In contrast, WO_3_T45Y, composed of 57.1% hexagonal hydrogen bronze and 42.9% hydrated orthorhombic WO_3_, showed a negligible optical response. Its color changed from pale yellow to green rather than blue, reflecting the superposition of the initial yellow absorption associated with the orthorhombic-rich component and the photoinduced blue coloration of the hexagonal hydrogen bronze fraction.

The measured values are consistent with previous reports on photoactive hexagonal WO_3_ nanostructures, although direct quantitative comparison remains limited by differences in sample geometry, irradiation conditions, and optical analysis methods [2,7,8]. Taken together, the results reveal a clear phase-dependent trend. Orthorhombic-rich samples are nearly inactive, while pure hexagonal phase NPs display a pronounced photochromic response, and samples containing hexagonal hydrogen bronze WO_3_ show the largest optical modulation. The superior performance of the hexagonal and hexagonal hydrogen bronze samples can be rationalized with literature evidence by considering the tunnel-like topology of the hexagonal WO_3_ polymorph. These channels have been reported to provide more accessible pathways for the insertion and migration of H^+^ during UV irradiation, thereby promoting the formation of mixed-valence W^5+^/W^6+^ centers responsible for IVCT and the characteristic blue coloration [14,15,34]. Moreover, the enhanced photochromic response of the samples containing the hexagonal hydrogen bronze is consistent with the presence of pre-existing structural protons and W^5+^ centers, which may lower the effective barrier for further photoinduced proton insertion and W^6+^→W^5+^ reduction upon UV irradiation [33,34,35]. Conversely, the orthorhombic phase possesses a denser and more compact framework, which is expected to limit proton intercalation and reduce the extent of photoinduced tungsten reduction [14,15]. These mechanistic considerations are consistent with the observed phase–response relationship.

### 2.4. Photochromic Behavior of PVP-WO_3_ Nanocomposites

WO_3_T75 was selected for incorporation into PVP because it combines pure hexagonal crystalline phase, a good photochromic response (ΔO.D._450nm_ = 0.15), and the highest synthesis yield among the temperature-dependent samples (72%). PVP was chosen as the host matrix because of its optical transparency in the UV-Vis region, its good film-forming ability, and its compatibility with metal oxide NPs. The polar character and oxygen permeability may also help to maintain a local environment favorable for the redox and proton transfer processes involved in WO_3_ photochromism, while previous studies have suggested that PVP can also support the reversibility of the coloration–bleaching cycle [36,37,38]. The photochromic response of the WO_3_/PVP films prepared with different NP loadings (1%, 5%, and 10% wt. relative to PVP) was first evaluated under 254 nm irradiation. The diffuse reflectance spectra at increasing irradiation times of WO_3_/PVP-10 are reported in Figure 5a, while the corresponding data for WO_3_/PVP-1 and WO_3_/PVP-5 are shown in Figure S16. The progressive decrease in reflectance and the approach to a plateau indicate that the coloration kinetics are retained after polymer incorporation.

As shown in Figure 5b, all nanocomposites underwent a visible color change after 25 min, evolving from transparent or white to blue. The magnitude of the response increased with WO_3_ loading: ΔO.D._450nm_ rose from 0.06 for WO_3_/PVP-1 to 0.16 for WO_3_/PVP-5 and 0.18 for WO_3_/PVP-10. The weak response observed at 1% is consistent with the low density of photoactive WO_3_ sites within the optical path. At higher loadings, the larger amount of WO_3_ provides a greater number of sites available for proton insertion, associated in the literature with the formation of mixed-valence W^5+^/W^6+^ centers through IVCT. The comparatively modest increase from 5% to 10% suggests, however, that the response began to approach a loading-dependent saturation regime under 254 nm irradiation.

Because 254 nm (UV-C) irradiation is of limited practical relevance for commercial UV application and is largely absent from terrestrial solar radiation [39], the nanocomposite films were also tested under UV-A at 365 nm. The time evolution of the diffuse reflectance spectra of WO_3_/PVP-10 under 365 nm irradiation wavelength is shown in Figure 6a, while the corresponding data for WO_3_/PVP-5 are reported in Figure S17. The WO_3_/PVP-10 sample reached the highest value of optical response of 0.21 after 25 min of irradiation (Figure 6b). The stronger response of the WO_3_/PVP-10 film at 365 nm indicates that a sufficiently high NP loading is required to obtain efficient near-UV activation.

The WO_3_/PVP-1 nanocomposite was not further considered at 365 nm because its response at the higher-energy excitation wavelength was weak and was expected to be below the useful measurement range. The samples at 10% loading show similar photochromic response when irradiated at the two distinct wavelengths 254 and 365 nm. This behavior can be ascribed to the higher PVP absorption at 254 nm that decreases the available irradiation for the WO_3_ NPs in the nanocomposite, in comparison with the negligible absorption of the PVP at 365 nm. In addition, enhanced light scattering and multiple absorption events within the film need to be taken into account, which can amplify the overall coloration efficiency in the near-UV range. The PVP may also contribute by promoting homogeneous NP dispersion and maintaining a local environment compatible with the hydration and redox processes involved in WO_3_ photochromism. Compared to bare WO_3_T75 films, which exhibited ΔO.D._450nm_ = 0.15 at 254 nm, the PVP nanocomposites at 10% loading not only preserved but actively enhanced the photochromic performance at both wavelengths. This improvement demonstrates that the PVP matrix plays an active role beyond mere mechanical support likely by increasing the NP dispersion in the organic matrix, stabilizing W^5+^ centers and facilitating proton exchange [28,29,40,41]. However, after irradiation, the PVP nanocomposites demonstrated a limited capacity to revert to their initial color, even when stored in the dark, and several days of storage are required to lose the acquired blue coloration. Further irradiation does not fully restore the initial coloration of the samples (Figure S19). Additional work is required to optimize the cyclability of the photochromic effect in order to reduce the reverse time and increase the coloration efficiency.

Overall, incorporation into PVP retains the photochromic activity of hexagonal WO_3_ and enables processable films responsive to both 254 nm and 365 nm irradiation, demonstrating that the polymer matrix is an effective platform for translating the optical response of WO_3_ NPs into solid-state photochromic coatings.

## 3. Experimental Procedure

### 3.1. Materials

Sodium tungstate dihydrate (Na_2_WO_4_·2H_2_O, 98%), hydrochloric acid (HCl, 37% wt.), 1-butanol (99.8%), absolute ethanol and polyvinylpyrrolidone K90 (PVP; MW = 360k) were purchased from Merck KGaA, (Darmstadt, Germany) and used as received, without further purification. Deionized water was used for all synthesis and washing procedures.

### 3.2. Synthesis of WO_3_ Nanoparticles by Precipitation Method

WO_3_ NPs were synthesized through an acid-induced sol–gel preparation method adapted from the procedure reported by Abbaspoor et al. [24], with modifications to the acidification conditions and thermal protocol. In a typical synthesis, 3.30 g of Na_2_WO_4_·2H_2_O (0.01 mol) was dissolved in 200 mL of deionized water under vigorous magnetic stirring. Concentrated HCl (37% wt.) was then added dropwise to the tungstate solution under continuous stirring. Acidification led to the formation of protonated tungstate species and tungstic acid intermediates, followed by condensation and dehydration to tungsten oxide. The reaction vessel was closed and maintained under vigorous stirring in a thermostatically controlled oil bath for 24 h. Two complementary synthetic protocols were investigated to distinguish the effects of acidification and reaction temperature.

In the acidification protocol, the amount of added HCl was varied from 1.7 mL (0.02 mol) up to 2.9 mL (0.035 mol), while the reaction temperature was maintained at 90° under gradual heating from room temperature. In the temperature-dependent series, the HCl amount was fixed at 2.9 mL, and the reactor was directly immersed in an oil bath preheated to 90 °C. After the initial temperature decrease caused by immersion, the bath was adjusted to the selected reaction temperature, ranging from 45 °C to 90 °C (Scheme 1).

The prepared NPs were named depending on the acid amount (n) and reaction temperature (t) as WO_3_An and WO_3_Tt, respectively. The sample labels and corresponding preparation conditions are summarized in Table 2.

After reaction, the products were recovered by centrifugation at 10,000 rpm for 30 min. The clear supernatant was discarded, and the precipitate was redispersed in fresh deionized water. Four cycles of centrifugation/precipitate redispersion in deionized water were carried out to remove residual sodium chloride, excess acid, and soluble precursor species. The purified products were dried overnight in an oven at 60 °C and collected as powders.

After drying, a double-colored powder, bluish and yellow, was observed for sample WO_3_T45. To separate the two contributions, the sample was fractionated by adjusting the centrifugation speed. The powder was dispersed in water and centrifuged at 3000 rpm for 5 min, recovering a yellow precipitate. The bluish supernatant was further centrifuged at higher speed, 10,000 rpm for 30 min, collecting the bluish powder. The two fractions were dried overnight at 60 °C, and labeled as WO_3_T45Y and WO_3_T45B, respectively.

### 3.3. Preparation of WO_3_/PVP Nanocomposite Films

WO_3_/PVP nanocomposites were prepared using a selected WO_3_ NP, WO_3_T75, at variable inorganic loading (1%, 5% and 10% wt.) relative to the mass of PVP, as summarized in Table 3.

For each nanocomposite sample, the required amount of WO_3_ NPs was dispersed in 2 mL of an H_2_O/EtOH mixture (1:3 *v*/*v*) and sonicated for 2 h. Separately, 1 g of PVP powder was dissolved in deionized water to a final volume of 20 mL and stirred for 1 h at room temperature. The WO_3_ suspension was then added to the PVP solution under continuous stirring, for further 24 h. Then, 500 μL of each dispersion was deposited onto cleaned glass substrates by drop casting and dried in oven at 70 °C for 2 h to obtain continuous nanocomposite films (Scheme 2).

### 3.4. Characterization Techniques

For optical characterization, WO_3_ NPs films were prepared by drop-casting 500 μL of a 100 mg/mL suspension of WO_3_ NPs in 1-butanol onto a 2.5 × 2.5 cm glass substrate. The deposited coatings were dried in an oven at 70 °C for 2h before measurements. Diffuse reflectance spectra were recorded using a Cary 5000 UV/Vis/NIR spectrophotometer (Agilent Technologies, Santa Clara, CA, USA) equipped with an integrating sphere. The optical response was analyzed using the Kubelka–Munk formalism. The measured reflectance, R, was converted into the Kubelka–Munk function:

$$F\left(R\right)=\frac{{\left(1-R\right)}^{2}}{2R}$$

The optical band gap, E_g_, was estimated by applying the Tauc relation [42]:

$${\left[F\left(R\right)h\nu \right]}^{\frac{1}{n}}=\beta \left(h\nu -E_{g}\right)$$

where hν is the photon energy, β a proportionality constant and n depends on the nature of the optical transition. For WO_3_, n = 2 was used, corresponding to an indirect allowed transition [43,44,45]. According to Brik et al. [46], E_g_ was obtained by tracing the tangent to the linear region of the [F(R)hν]^1/2^ versus hν plot, evaluating the effect of background absorption on the extracted values by considering the intersection of the fitted absorption edge line with the experimental background. The determination of the optical band gap of WO_3_ by means of a Tauc analysis requires caution, particularly when different WO_3_ polymorphs, mixed-phase samples, and defect-related absorption features are considered, as in our case. We therefore refer to the values obtained from the Tauc analysis as apparent optical band-gap values throughout this work. The primary purpose of Eg estimation in the present work is not to provide an absolute and definitive determination of the fundamental electronic band gap of each WO_3_ sample. Rather, the extracted values are used as a consistent comparative parameter to assess differences in the optical absorption edge among the investigated samples.

Particle morphology was investigated by transmission electron microscopy (TEM) using a JEOL JEM1011 microscope, operating at an accelerating voltage of 100 kV. The dried powders were dispersed in ethanol (1 mg/mL) and sonicated for 1 h to achieve uniform dispersion. Then, 3 μL of the diluted suspension was drop-cast onto a 400-mesh amorphous carbon-coated Cu grid, allowed to dry, and then measured by TEM. X-ray powder diffraction (XRPD) patterns were collected by a Rigaku RINT2500 rotating-anode diffractometer (50 kV, 200 mA) equipped with a Rigaku D/teX Ultra silicon strip detector. A Johansson Ge(111) crystal was employed to select monochromatic Cu Kα_1_ radiation (λ = 1.54056 Å). Measurements were carried out in transmission mode by loading the powders into 0.5 mm Lindemann capillaries. XRPD data were collected over the 2θ range of 10–100° using a step size of 0.02° and a fixed counting time of 2 s per step. Qualitative phase analysis was performed using QUALX 2.0 software. After the qualitative phase analysis, the XPRD patterns were analyzed by using a whole-profile Rietveld-based fitting program QUANTO [47]. The quality of the Rietveld refinements was evaluated separately from the refined profiles and from the difference curves, together with the corresponding Rp(%), Rwp(%), and χ^2^ values.

The photochromic response was studied by irradiating the solid-state films with a 254 nm and 365 nm 8W UV lamps (intensity: 0.2 mW/cm^2^) placed at 10 cm from the sample for different exposure times. To compare the photochromic behavior of the WO_3_ films and nanocomposites, the variation in the optical density after irradiation ΔO.D. was considered as a performance indicator:

$$\Delta O.D.=\log\frac{R_{b}}{R_{a}}$$

which is defined as the logarithm of the ratio between the reflectance before (R_b_) and after irradiation (R_a_) at a specific wavelength [7]. In this work λ = 450 nm was selected, as it corresponds to the blue region of the visible spectrum, where the photoinduced coloration of WO_3_ produces the strongest contrast detectable to the human eye, making it the most informative wavelength for evaluating performance in smart window and optical device applications.

## 4. Conclusions

This work establishes a clear relationship between the preparation conditions, crystalline phase composition, and photochromic response of WO_3_ NPs obtained through a simple acid-induced sol–gel precipitation route. Within the investigated acidification range, increasing the amount of HCl produced only limited changes in morphology and hydrogenation state. The resulting materials remained predominantly orthorhombic or hydrated orthorhombic, retaining a mainly sheet-like morphology, with apparent optical band gaps of approximately 2.4 eV, and showed negligible photochromic response, with ΔO.D._450nm_ values of about 0.008.

By contrast, the heating conditions exerted a substantially stronger influence on NP formation. Rapid heating promoted the formation of rod-like nanostructures with hexagonal phase, while decreasing the reaction temperature progressively promoted hydrogen bronze phases. Pure hexagonal phase was obtained at 75 °C, whereas hexagonal hydrogen bronze contributions emerged at 60 °C and 45 °C. The phase composition was directly reflected in the photochromic behavior: samples containing hexagonal and hexagonal hydrogen bronze WO_3_ reached ΔO.D._450nm_ values between 0.15 and 0.20 under 254 nm irradiation, compared with the nearly inactive orthorhombic materials. These results identify crystalline phase and the presence of hydrogen as the main structural parameters associated with the photochromic response in the investigated series.

Among the investigated formulations, WO_3_T75 provided the most favorable combination of phase purity, photochromic response, and synthesis yield. Its incorporation into PVP yielded nanocomposite films that retained a pronounced optical response under both 254 and 365 nm irradiation. The magnitude of the coloration increased with nanoparticle loading, reaching ΔO.D._450nm_ of 0.18 at 254 nm and 0.21 at 365 nm for the 10 wt.% film. These results demonstrate that polymer embedding preserves the functionality of the hexagonal WO_3_ NPs and provides a suitable route for their conversion into solid-state photochromic coatings.

Overall, the study shows that temperature is the most effective synthetic lever for directing WO_3_ phase selection in this precipitation system. Control of the reaction temperature and heating profile enables the preparation of hexagonal and hexagonal hydrogen bronze NPs without structure-directing additives or post-synthetic phase conversion treatments. Their subsequent integration into PVP extends the photochromic response to a processable film format, providing a practical basis for the development of WO_3_-based coatings for smart windows, optical displays, and light-responsive devices.

## Figures and Tables

**Figure 1:**
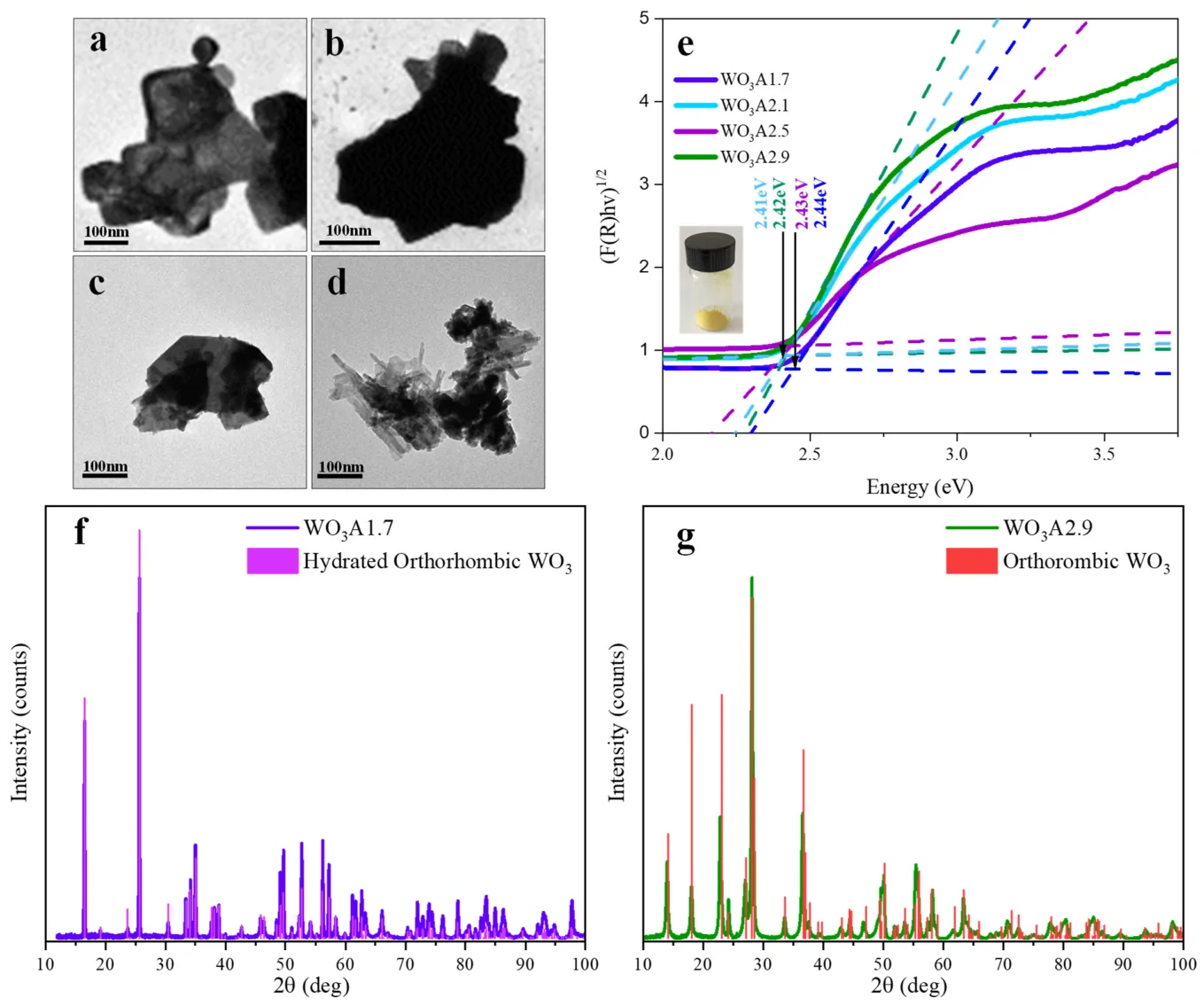
TEM micrographs of WO_3_ NPs synthesized by gradually heating the reaction mixture up to 90 °C using increasing amounts of HCl: (**a**) WO_3_A1.7, (**b**) WO_3_A2.1, (**c**) WO_3_A2.5, and (**d**) WO_3_A2.9. (**e**) Tauc plots derived from Kubelka–Munk function determined from diffuse reflectance spectra; the inset shows a representative picture of WO_3_A1.7 yellow-colored powder. Experimental XRPD profiles and reference XRPD patterns of (**f**) WO_3_A1.7, JCPD Card No. 00-900-4174 and (**g**) WO_3_A2.9, JCPD Card No. 00-901-4468.

**Figure 2:**
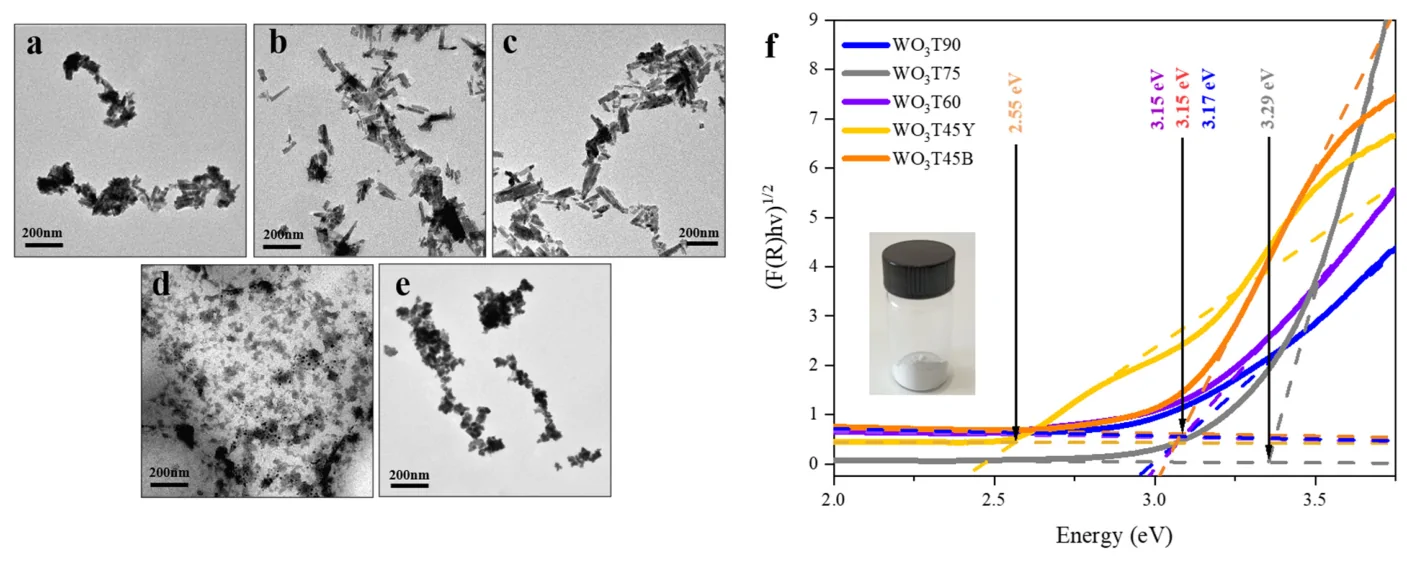
TEM images of WO_3_ NPs synthesized at different reaction temperatures: (**a**) WO_3_T90, (**b**) WO_3_T75, (**c**) WO_3_T60, (**d**) the bluish WO_3_T45B fraction, and (**e**) the yellow WO_3_T45Y fraction of the WO_3_T45 sample. (**f**) Tauc plots obtained from Kubelka–Munk function, as determined from diffuse reflectance spectra for determination of the apparent optical band gaps. The inset shows the macroscopic appearance of WO_3_T75 powder.

**Figure 3:**
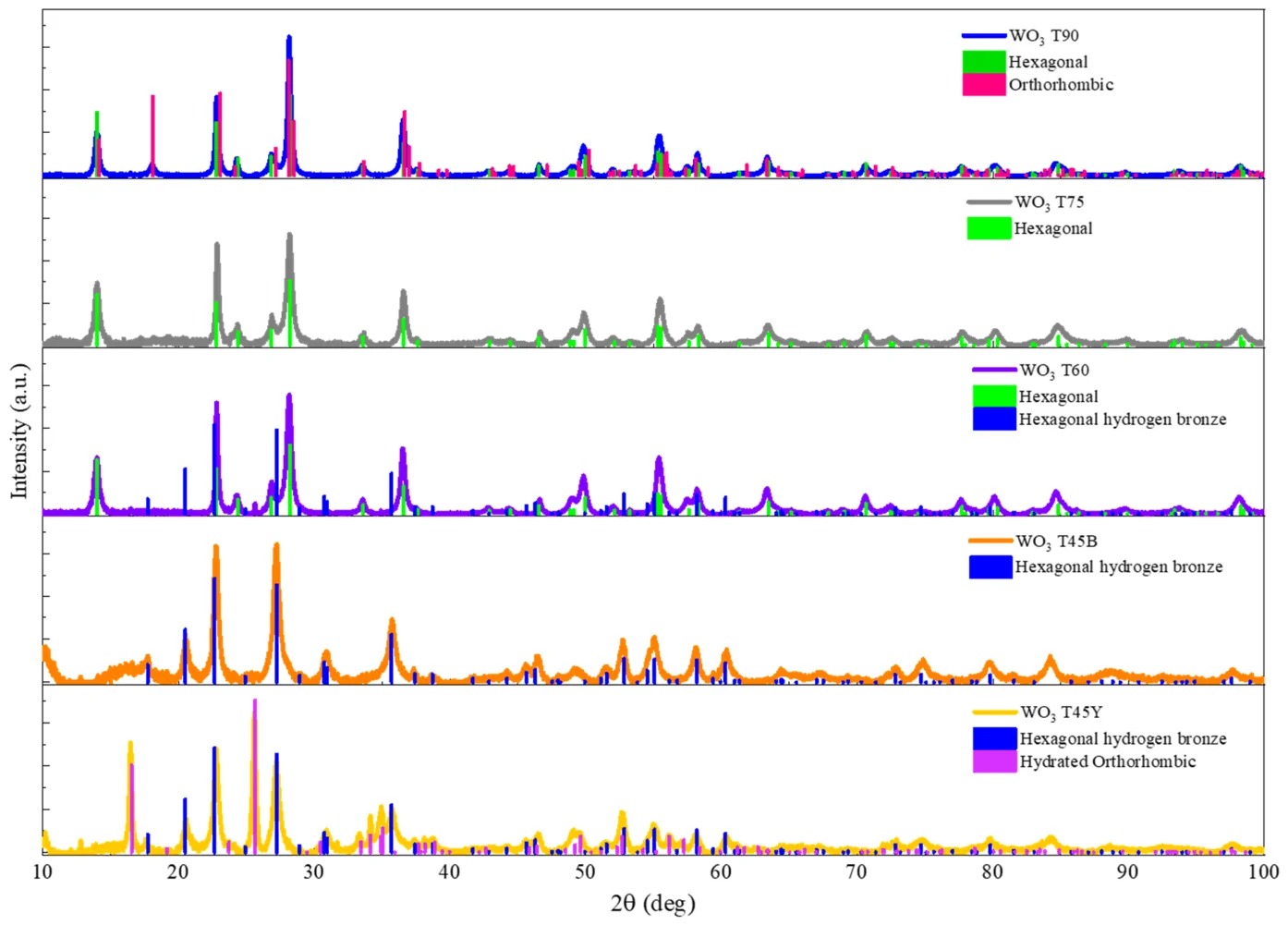
XRPD patterns of WO_3_T90, WO_3_T75, WO_3_T60, WO_3_T45B and WO_3_T45Y, together with the reference reflections assigned to hexagonal, orthorhombic, hexagonal hydrogen bronze, and hydrated orthorhombic WO_3_ phases.

**Figure 4:**
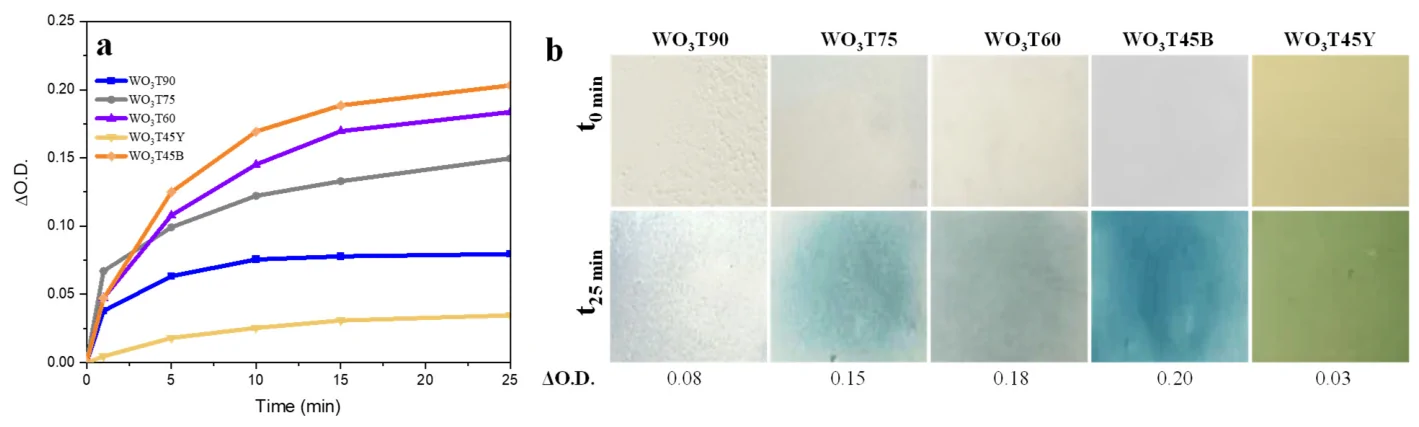
(**a**) ΔO.D._450nm_ kinetic of WO_3_T series. (**b**) Pictures of films of WO_3_ NPs synthesized at different temperature before (t_0min_) and after 25 min (t_25min_) of irradiation under UV light at λ = 254 nm, together with the corresponding ΔO.D._450nm_ values.

**Figure 5:**
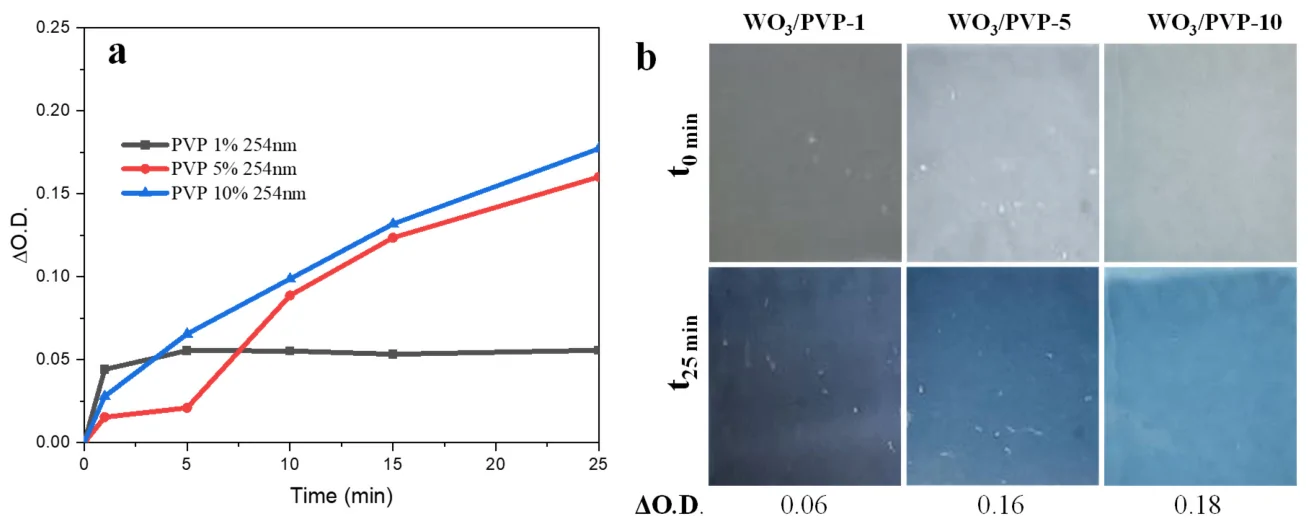
(**a**) ΔO.D._450nm_ kinetic of WO_3_/PVP samples irradiated at 254 nm. (**b**) Photographs of WO_3_/PVP-1, WO_3_/PVP-5, and WO_3_/PVP-10 nanocomposite films before (t_0min_) and after 25 min (t_25min_) of irradiation by an UV lamp at 254 nm, together with the corresponding ΔO.D._450nm_ values.

**Figure 6:**
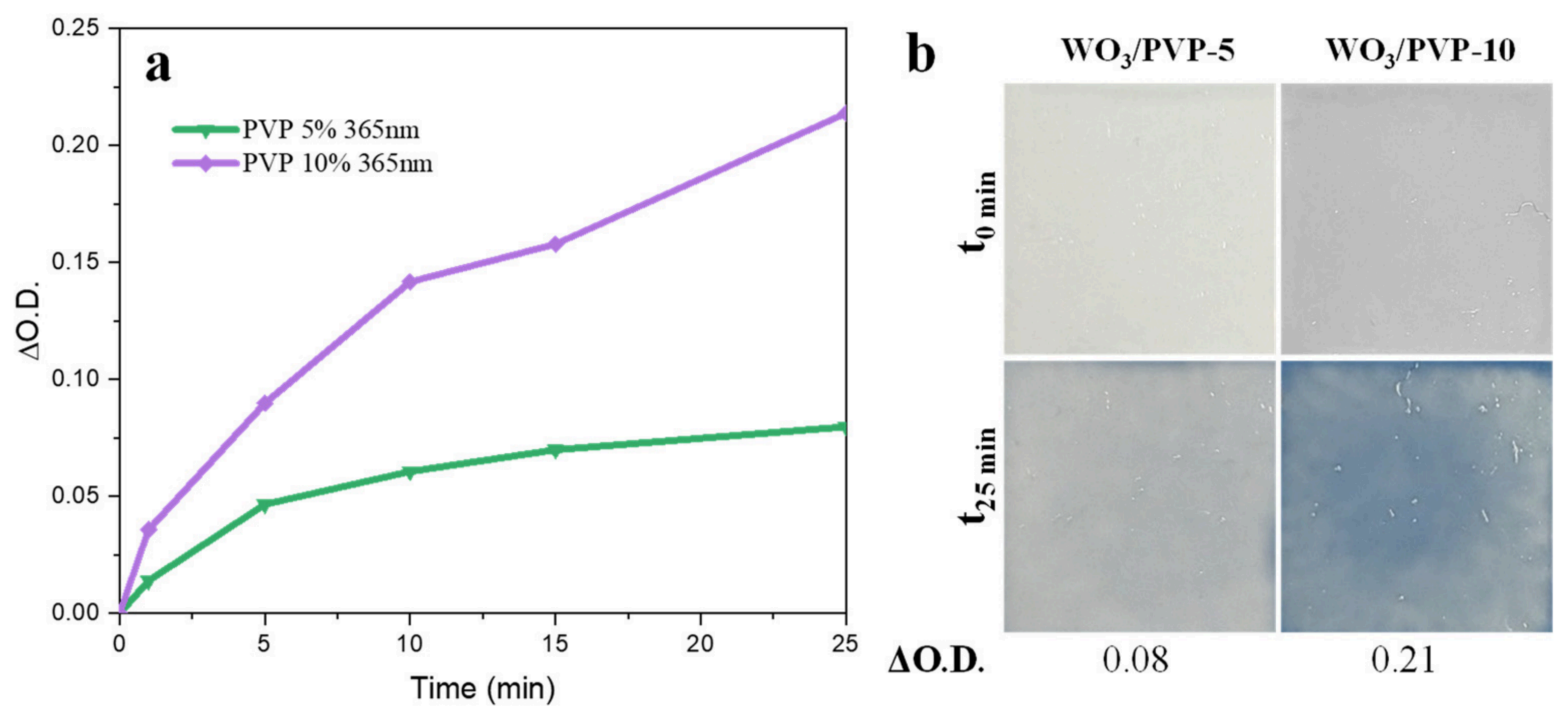
(**a**) ΔO.D._450nm_ kinetic of WO_3_/PVP samples irradiated at 365 nm. (**b**) Photographs of WO_3_/PVP-5 and WO_3_/PVP-10 before (t_0min_) and after 25 min (t_25min_) at 365 nm, together with the corresponding ΔO.D._450nm_ values.

**Scheme 1:**
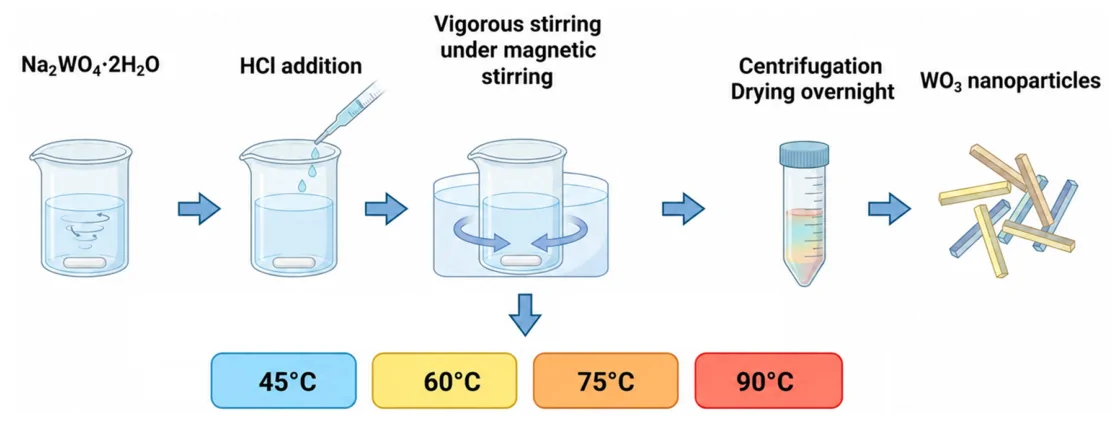
Schematic overview of the temperature-controlled synthesis of WO_3_ NPs.

**Scheme 2:**
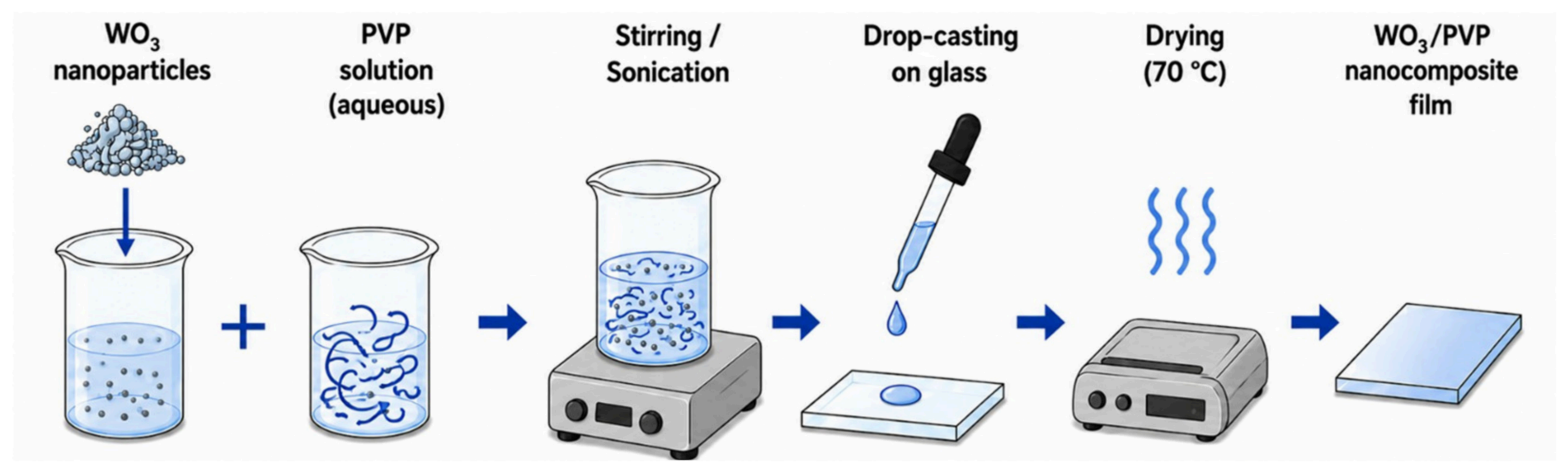
Sketch of WO_3_/PVP nanocomposite sample preparation.

**Table 1:** Reaction yield, calculated Eg and crystallographic data of WO_3_ NPs synthesized at different temperatures.

Sample	Yield (%)	E_g_ (eV)	WO_3_ Crystalline Phase	Phase Fraction (%)	JCPDS Card
WO_3_T90	58	3.17 ± 0.03	Hexagonal	75.7 ± 0.5	00-100-4057
			Orthorhombic	24.3 ± 0.6	00-901-4468
WO_3_T75	72	3.29 ± 0.03	Hexagonal	100 ± 1.9	00-100-4057
WO_3_T60	59	3.15 ± 0.03	Hexagonal	87.1 ± 0.2	00-100-4057
			Hexagonal hydrogen bronze	12.9 ± 0.9	04-026-3011
WO_3_T45B	13	3.15 ± 0.03	Hexagonal hydrogen bronze	100 ± 1.9	04-026-3011
WO_3_T45Y	38	2.55 ± 0.02	Hexagonal hydrogen bronze	57.1 ± 0.7	04-026-3011
			Hydrated orthorhombic	42.9 ± 1.9	04-011-6930

**Table 2:** Sample nomenclature and synthesis conditions of the WO_3_ NPs prepared following the acidification and temperature protocols.

Sample	HCl Added (mL)	HCl Amount (mol)	Reaction Temperature (°C)	Heating Protocol
WO_3_A1.7	1.7	0.020	90	Gradual heating from room temperature
WO_3_A2.1	2.1	0.025	90	Gradual heating from room temperature
WO_3_A2.5	2.5	0.030	90	Gradual heating from room temperature
WO_3_A2.9	2.9	0.035	90	Gradual heating from room temperature
WO_3_T90	2.9	0.035	90	Direct immersion in a preheated oil bath
WO_3_T75	2.9	0.035	75	Direct immersion in a preheated oil bath
WO_3_T60	2.9	0.035	60	Direct immersion in a preheated oil bath
WO_3_T45	2.9	0.035	45	Direct immersion in a preheated oil bath

**Table 3:** Composition of the WO_3_/PVP nanocomposites.

Sample Code	WO_3_ Mass (mg)	PVP Mass (g)	Loading (wt.% Relative to PVP)
WO_3_/PVP-1	10	1.0	1
WO_3_/PVP-5	50	1.0	5
WO_3_/PVP-10	100	1.0	10

## Data Availability

Data is contained within the article or Supplementary Materials. The original contributions presented in this study are included in the article/Supplementary Materials. Further inquiries can be directed to the corresponding authors.

## Supplementary Materials

The following supplementary materials can be downloaded at https://www.mdpi.com/article/10.3390/molecules31193460/s1: The supplementary file contains the following figures and tables. Table S1: XRPD quantitative analysis values and cell parameters of the WO_3_A series synthesized at 90 °C; Table S2: XRPD quantitative analysis values and cell parameters of WO_3_A2.9R_75 and WO_3_A2.9R_60; Table S3: XRPD quantitative analysis values and cell parameters of the WO_3_T series; Figure S1: Rietveld refinement plot of the WO_3_A1.7 sample, fitted with a hydrated orthorhombic phase; Figure S2: Rietveld refinement plot of the WO_3_A2.9 sample, fitted with an orthorhombic phase; Figure S3: Experimental XRD profiles and reference XRPD patterns of (a) WO_3_A2.1 (JCPD Card No. 00-900-4174) and (b) WO_3_A2.5 (JCPD Card No. 00-901-4468); Figure S4: Rietveld refinement plot of the WO_3_A2.1 sample, fitted with a hydrated orthorhombic phase; Figure S5: Rietveld refinement plot of the WO_3_A2.5 sample, fitted with an orthorhombic phase; Figure S6: Experimental XRD profiles and reference XRPD patterns of (a) WO_3_A2.9R_75 and (b) WO_3_A2.9R_60; Figure S7: Rietveld refinement plot of the XRD profile of the WO_3_A2.9R_75 sample; Figure S8: Rietveld refinement plot of the XRD profile of the WO_3_A2.9R_60 sample; Figure S9: Rietveld refinement plot of the XRD profile of the WO_3_T90 sample; Figure S10: Rietveld refinement plot of the XRD profile of the WO_3_T75 sample; Figure S11: Rietveld refinement plot of the XRD profile of the WO_3_T60 sample; Figure S12: Rietveld refinement plot of the XRD profile of the WO_3_T45B sample; Figure S13: Rietveld refinement plot of the XRD profile of the WO_3_T45Y sample; Figure S14: Size distribution analysis of (a) WO_3_T90, (b) WO_3_T75, and (c) WO_3_T60; Figure S15: Photochromic kinetic diffuse reflectance spectra of WO_3_A1.7 during 80 min of irradiation at λ = 254 nm; Figure S16: Photochromic kinetic diffuse reflectance spectra of (a) WO_3_T90, (b) WO_3_T75, (c) WO_3_T60, (d) WO_3_T45B, and (e) WO_3_T45Y during 25 min of irradiation at λ = 254 nm; Figure S17: Photochromic kinetic diffuse reflectance spectra of (a) WO_3_/PVP-1, (b) WO_3_/PVP-5, and (c) WO_3_/PVP-10 during 25 min of irradiation at λ = 254 nm; Figure S18: Photochromic kinetic diffuse reflectance spectra of (a) WO_3_/PVP-5 and (b) WO_3_/PVP-10 during 25 min of irradiation at λ = 365 nm; Figure S19: Self-bleaching kinetics of the WO_3_/PVP-10 sample irradiated at (a) 254 nm and (b) 365 nm, and ΔO.D._450nm_ bleaching kinetics after irradiation at (c) 254 nm and (d) 365 nm. Following an initial 25 min irradiation, the samples were allowed to recover in the dark for 18 days before a second 25 min irradiation. Figures S1, S2, S4, S5, S6–S13: Rietveld refinements of the XRPD diffractograms of WO_3_A1.7, WO_3_A2.9, WO_3_A2.1, WO_3_A2.5, WO_3_T90, WO_3_T75, WO_3_T60, WO_3_T45B, WO_3_T45Y, WO_3_2.9R_75, WO_3_2.9R_60 and XRPD diffractograms of WO_3_2.9R_75 and WO_3_2.9R_60, respectively; Figure S3: XRPD profiles of WO_3_A2.1 and WO_3_A2.5; Figure S14: size distribution of samples WO_3_T90, WO_3_T75 and WO_3_T60; Figures S15–S18: Photochromic kinetic diffuse reflectance spectra of WO_3_A1.7, WO_3_T series, WO_3_/PVP-1, WO_3_/PVP-5 and WO_3_/PVP-10 irradiated at 254 nm, and WO_3_/PVP-5 and WO_3_/PVP-10 irradiated at 365 nm, respectively. Figure S19: Self-bleaching kinetic of WO_3_/PVP-10 sample irradiated at 254 nm and 365 nm. Tables S1–S3: XRPD quantitative analysis values and cell parameters of WO_3_A series, WO_3_T series, WO_3_2.9R_75 and WO_3_2.9R_60, respectively.
