# Supplementary material for: Phase Engineering of WO3 Nanoparticles Towards Efficient Photochromic Nanocomposites
Source: Molecules. 2026 Sep 28;31(19):3460. doi: 10.3390/molecules31193460 (PMC13666272; doi:10.3390/molecules31193460)
Supplement: Supplementary file 1 [file molecules-31-03460-s001.zip › molecules-4509635-supplementary.pdf]

molecules

SUPPORTING INFORMATIONS

MDPI

Phase Engineering of WO_3 Nanoparticles towards Efficient Photochromic Nanocomposites

Nicola Caggiano,[1,†] Carlo Nazareno Dibenedetto,[2,†] Elisabetta Fanizza,[1] Pierluigi Lasala,[1] Annamaria Panniello,[2] Teresa Sibillano,[3] Cinzia Giannini,[3] Maria Lucia Curri,[2,*] Marinella Striccoli[1,*]

[1] Dipartimento di Chimica, Università degli Studi di Bari, Via Orabona 4, 70126 Bari, Italy
[2] CNR-Istituto per i Processi chimico Fisici (CNR-IPCF), Via Orabona 4, 70126 Bari, Italy
[3] CNR-Istituto di Cristallografia (CNR-IC), Via Amendola, 122/O, 70126 Bari, Italy
* Correspondence: carlonazareno.dibenedetto@cnr.it , marinella.striccoli@cnr.it
† These authors contributed equally to this work

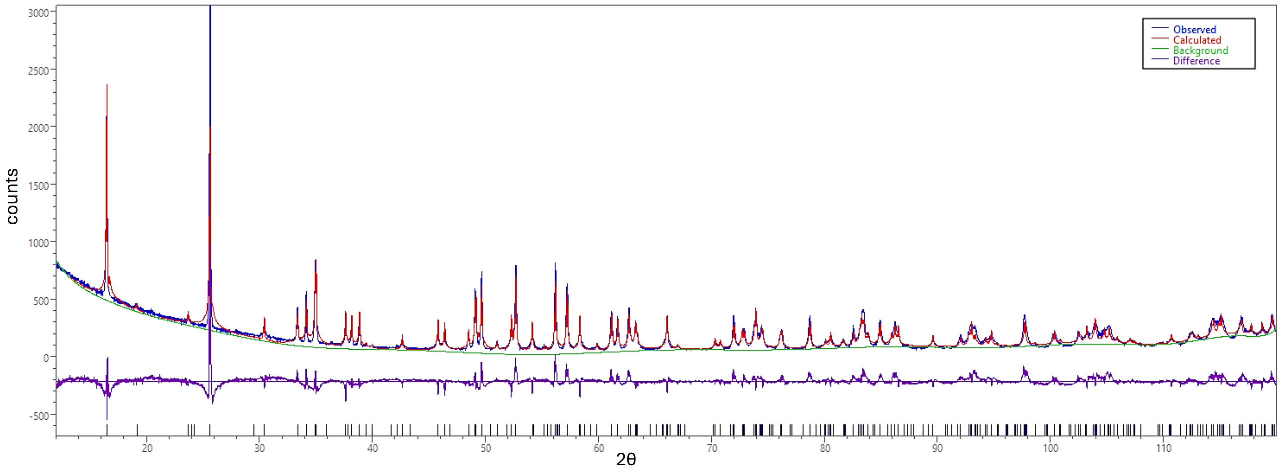

Figure S1. Rietveld refinement plot of the WO_3A1.7 sample well-fitting with a hydrated orthorhombic phase.

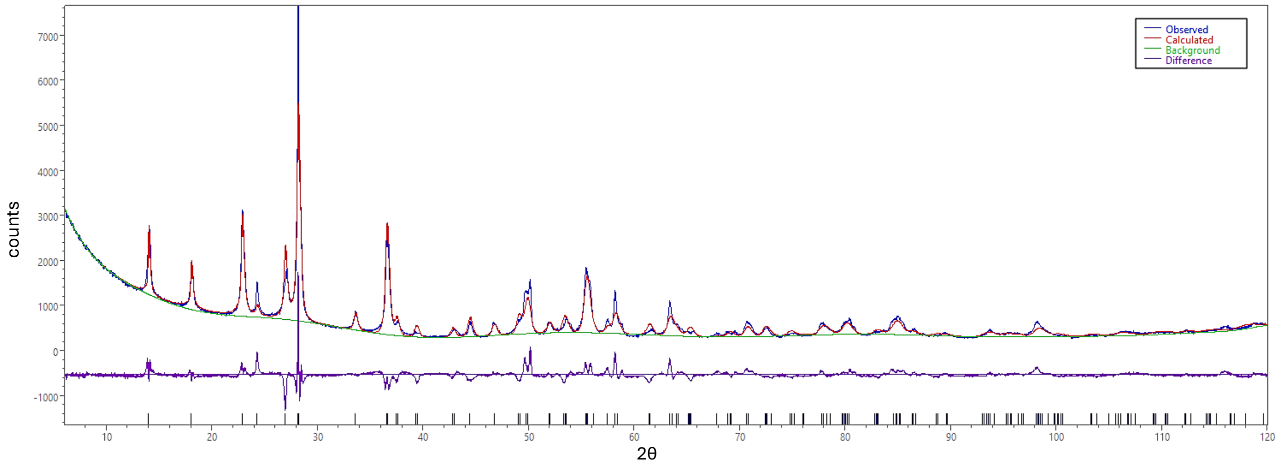

Figure S2. Rietveld refinement plot of the WO_3A2.9 sample well-fitting with an orthorhombic phase.

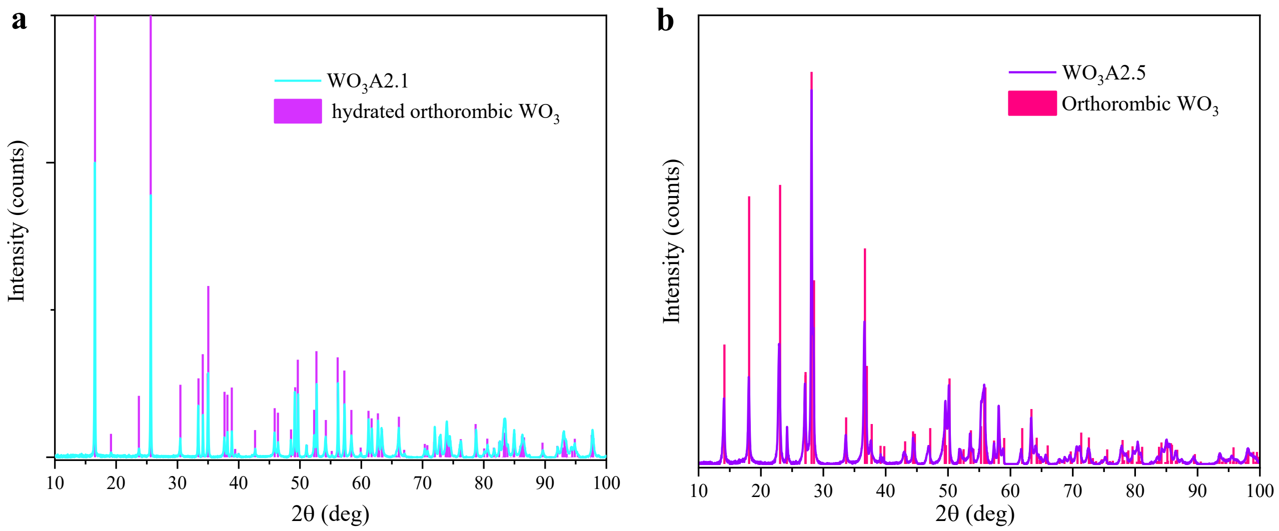

Figure S3. Experimental XRD profiles and reference XRPD patterns of (a) WO_3A2.1, JCPD Card No. 00-900-4174 and (b) WO_3A2.5, JCPD Card No. 00-901-4468

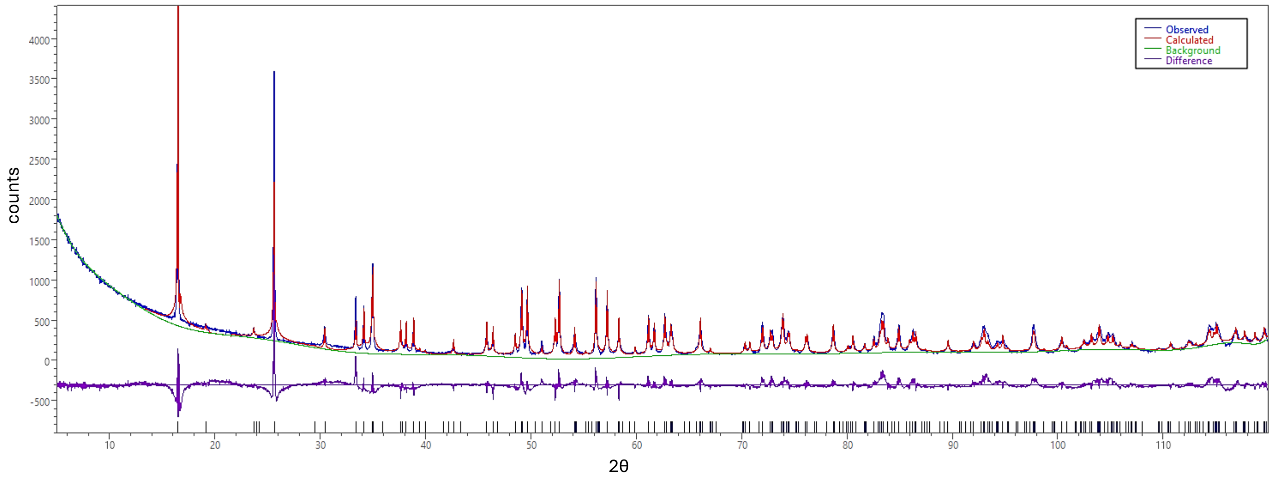

Figure S4. Rietveld refinement plot of the WO_3A2.1 sample well-fitting with a hydrated orthorhombic phase.

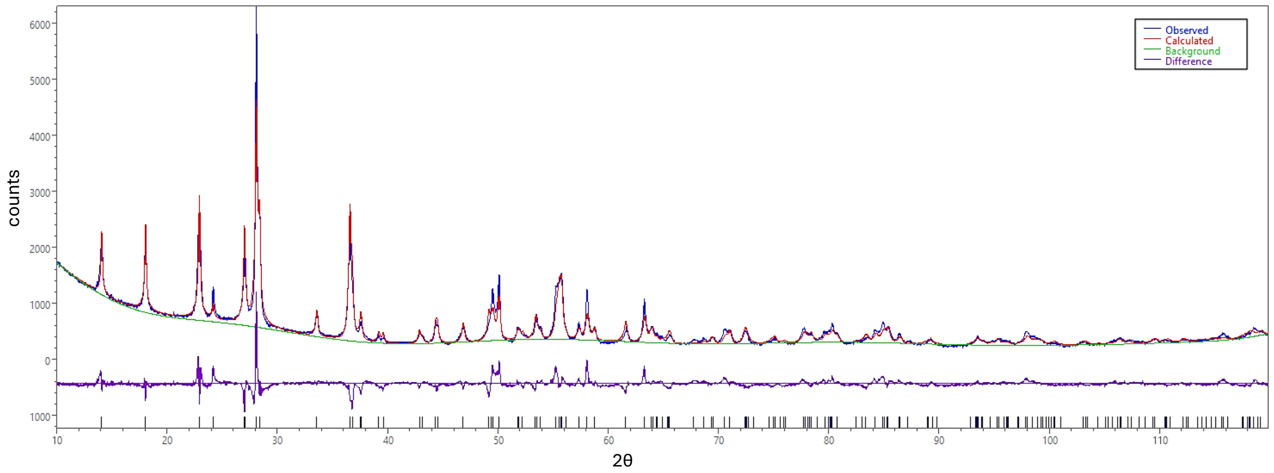

Figure S5. Rietveld refinement plot of the WO_3A2.5 sample well-fitting with an orthorhombic phase.

Sample	WO_3 crystalline phase	Rwp (%)	Rp (%)	χ^2	a (Å)	b (Å)	c (Å)	Unit-cell volume (Å³)
**WO3 A1.7**	Hydrated orthorhombic	14.784	10.971	4.242	5.24399	10.72528	5.13221	288.65
**WO3 A2.1**	Hydrated orthorhombic	13.859	9.368	5.516	5.24607	10.72528	5.13216	288.83
**WO3 A2.5**	orthorhombic	9.062	6.355	4.438	7.35276	12.56886	7.75112	716.33
**WO3 A2.9**	orthorhombic	9.380	6.181	5.977	7.32152	12.62291	7.76541	717.67

Table S1. XRPD quantitative analysis values and cell parameters of WO_3A series synthetized at 90°C.

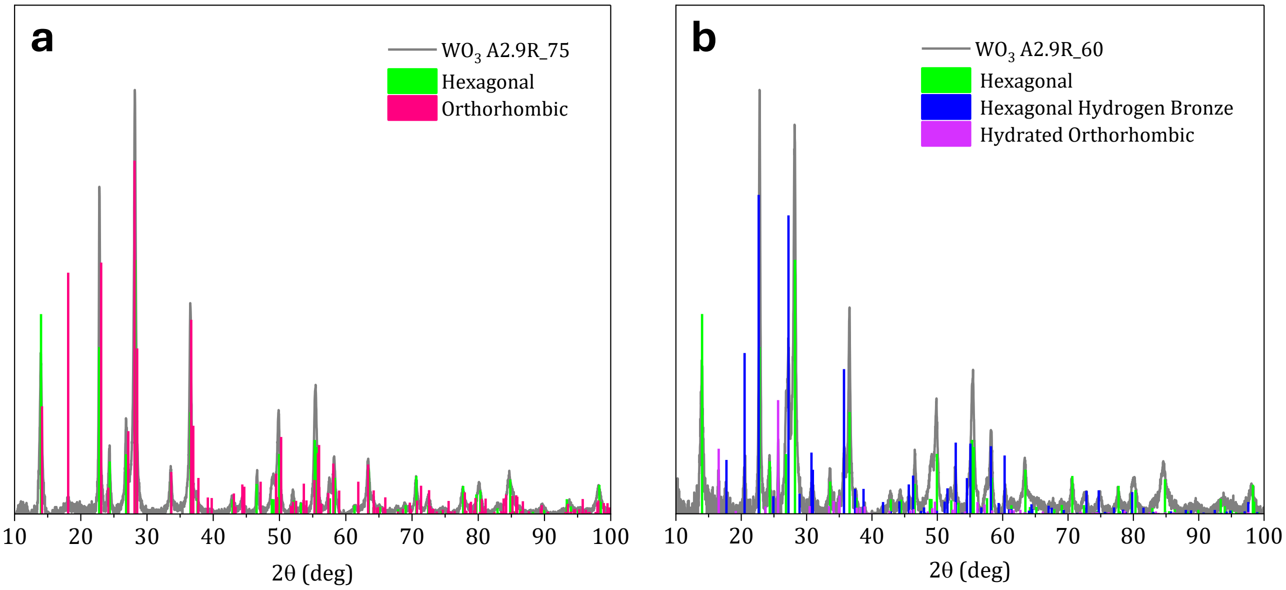

Figure S6. Experimental XRD profiles and reference XRPD patterns of (a) WO_3A2.9R_75 and (b) WO_3A2.9R_60.

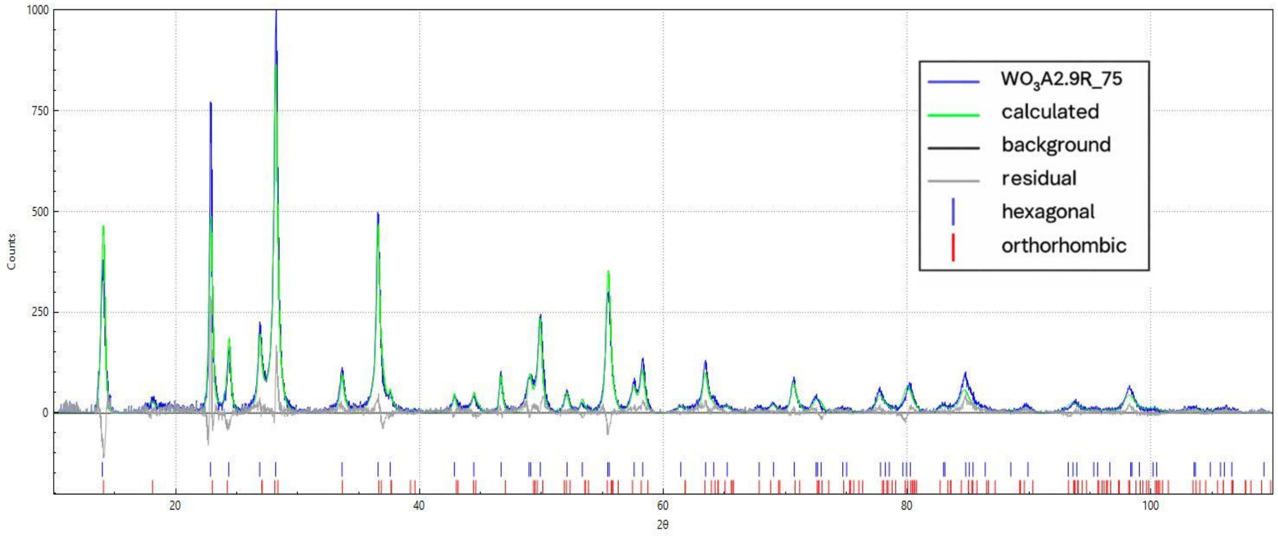

Figure S7. Rietveld refinement plot of XRD profile of WO_3A2.9R_75 sample.

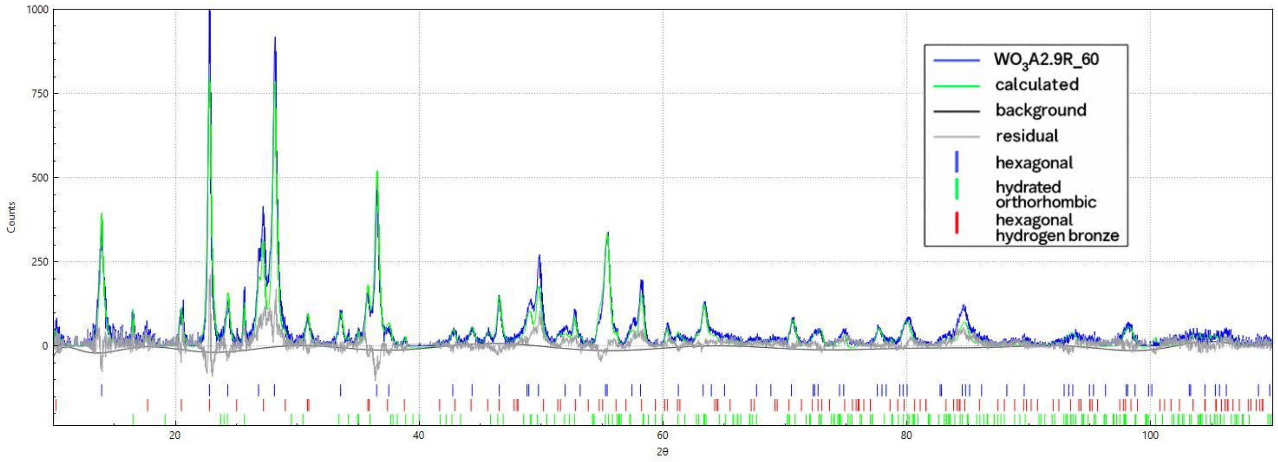

Figure S8. Rietveld refinement plot of XRD profile of WO_3A2.9R_60 sample.

Sample	WO_3 crystalline phase	Rwp (%)	Rp (%)	χ^2	a (Å)	b (Å)	c (Å)	Unit-cell volume (Å³)
WO_3A2.9R_75	Hexagonal 63%	35.564	23.75	3.880	7.30205	7.30205	3.88728	179.50
	Orthorhombic 37%				7.33982	12.56377	7.71717	711.65
WO_3A2.9R_60	Hexagonal 50%	42.243	30.848	7.730	7.32043	7.32043	3.89815	180.91
	Hexagonal hydrogen bronze 21 %				10.00110	10.00110	3.89839	337.68
	Hydrated orthorhombic 29 %				5.2404	10.71633	5.13028	288.11

Table S2. XRPD quantitative analysis values and cell parameters of WO_3A2.9R_75 and WO3A2.9R_60.

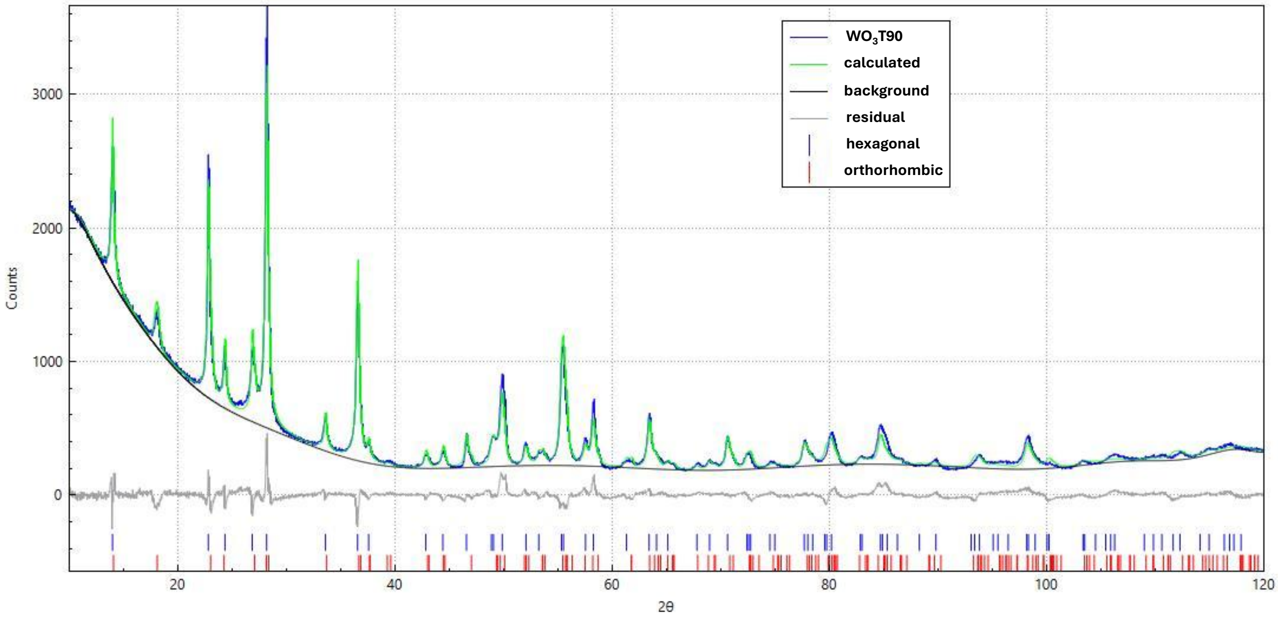

Figure S9. Rietveld refinement plot of XRD profile of WO_3T90 sample.

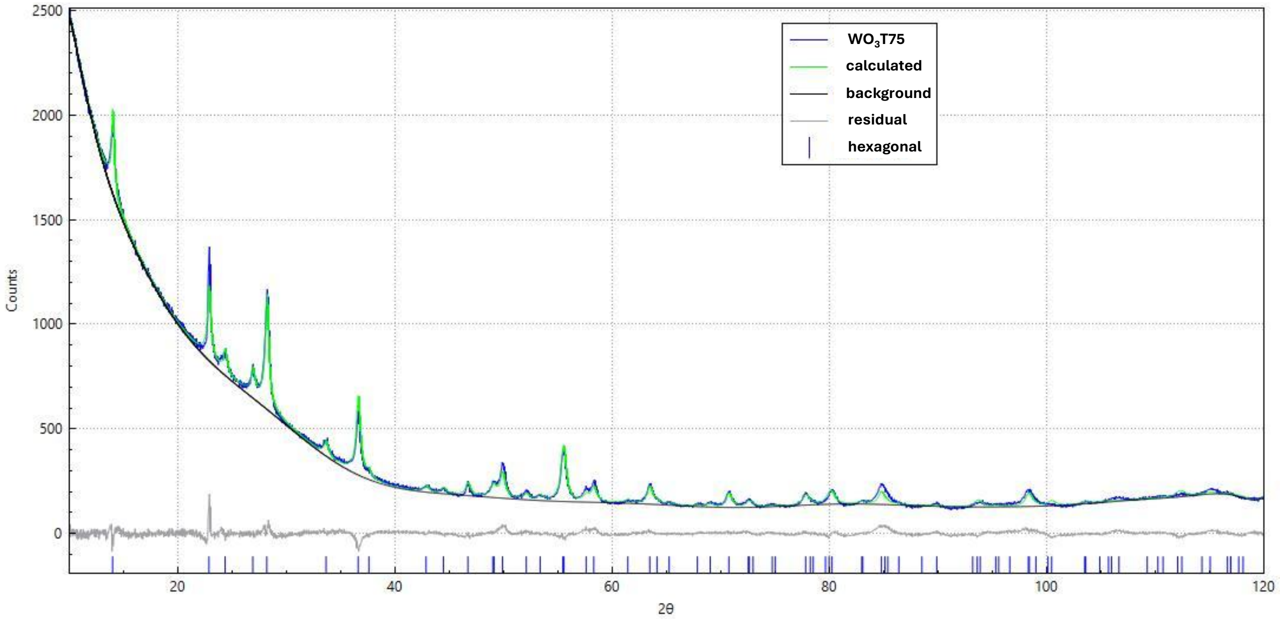

Figure S10. Rietveld refinement plot of XRD profile of WO_3T75 sample.

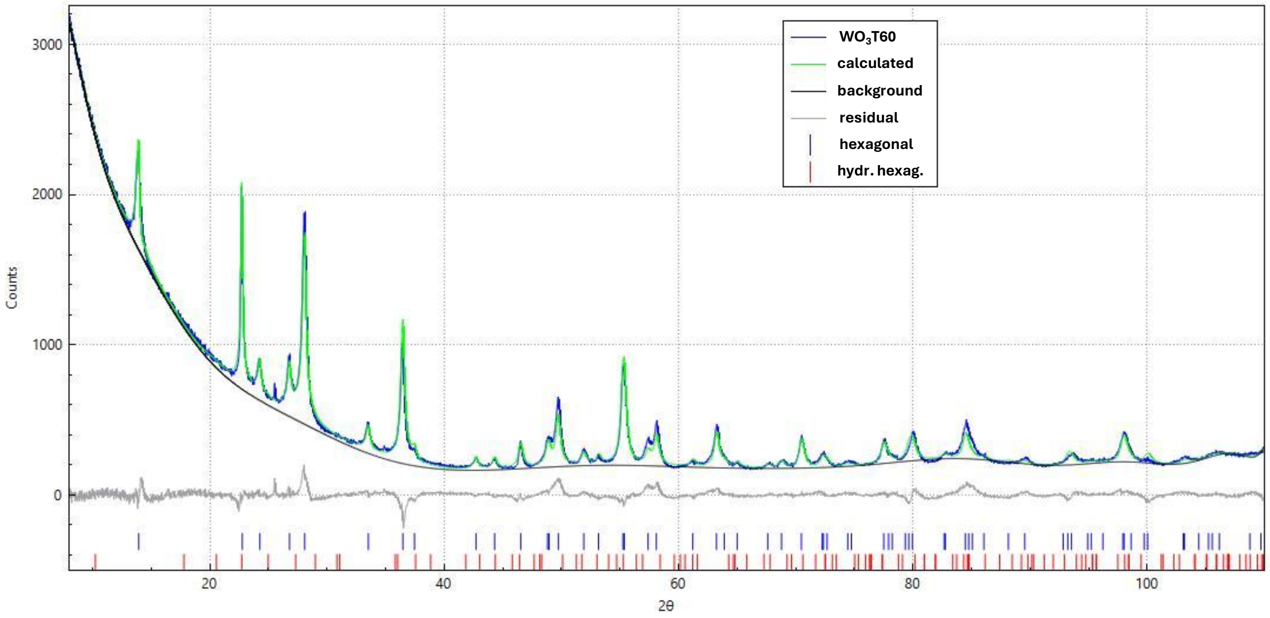

Figure S11. Rietveld refinement plot of XRD profile of WO_3T60 sample.

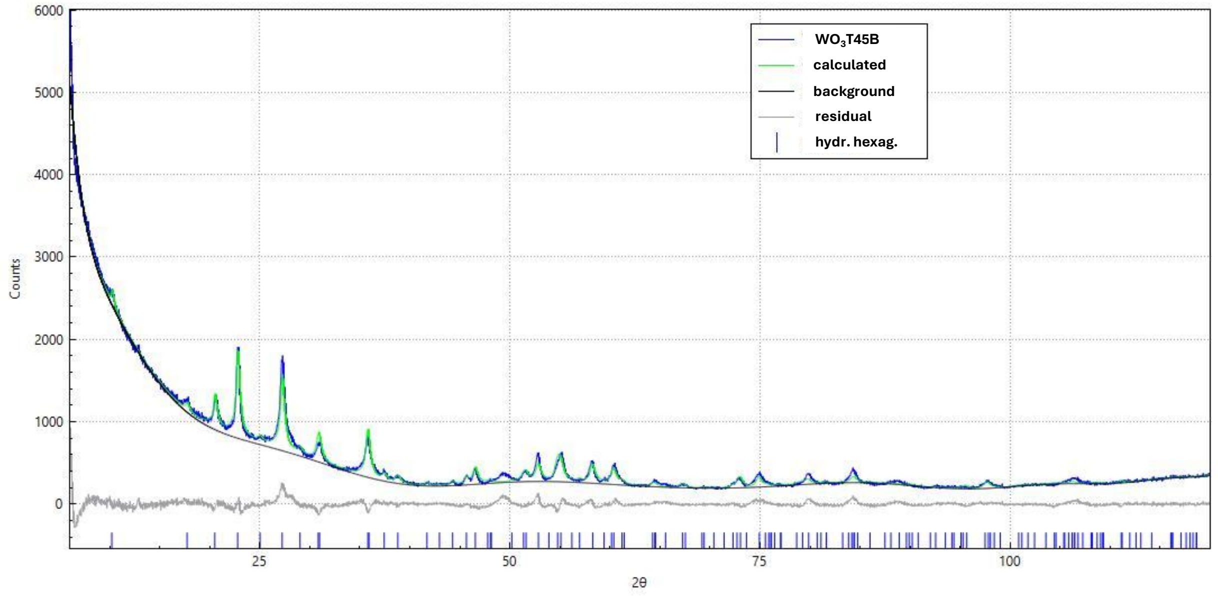

Figure S12. Rietveld refinement plot of XRD profile of WO_3T45B sample.

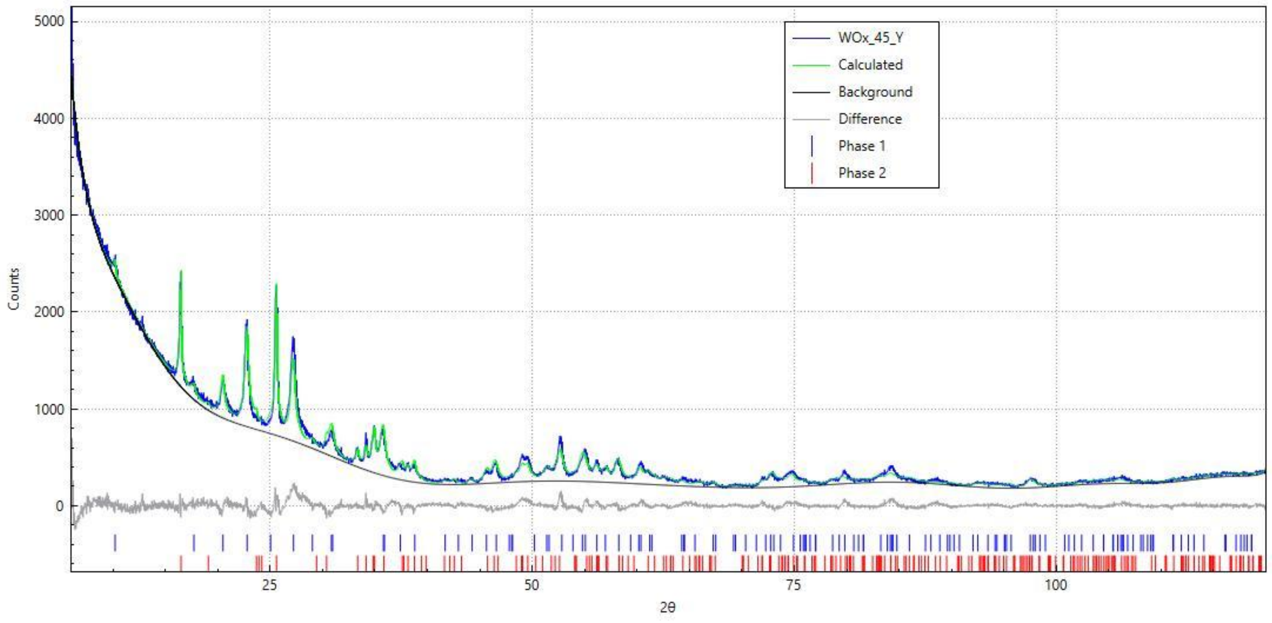

Figure S13. Rietveld refinements plot of XRD profile of WO_3T45Y sample.

Sample	WO_3 crystalline phase	Rwp (%)	Rp (%)	χ^2	a (Å)	b (Å)	c (Å)	Unit-cell volume (Å³)
WO_3T90	Hexagonal	5.764	4.013	1.590	7.30814	7.30814	3.89674	180.24
	Orthorhombic				7.33249	12.57550	7.72255	712.09
WO_3T75	Hexagonal	3.495	2.184	0.462	7.30427	7.30427	3.88643	179.57
WO_3T60	Hexagonal	5.109	3.306	1.316	7.32511	7.32511	3.89942	181.20
	Hexagonal hydrogen bronze				9.95788	9.95788	3.90724	335,53
WO_3T45Y	Hexagonal hydrogen bronze	3.903	5.388	1.721	9.99677	9.99677	3.89660	337.24
	Hydrated orthorhombic				5.24566	10.73886	5.14831	290.02
WO_3T45B	Hexagonal hydrogen bronze	5.969	4.239	2.054	9.99280	9.99280	3.89869	337.15

Table S3. XRPD quantitative analysis values and cell parameters of WO_3T series.

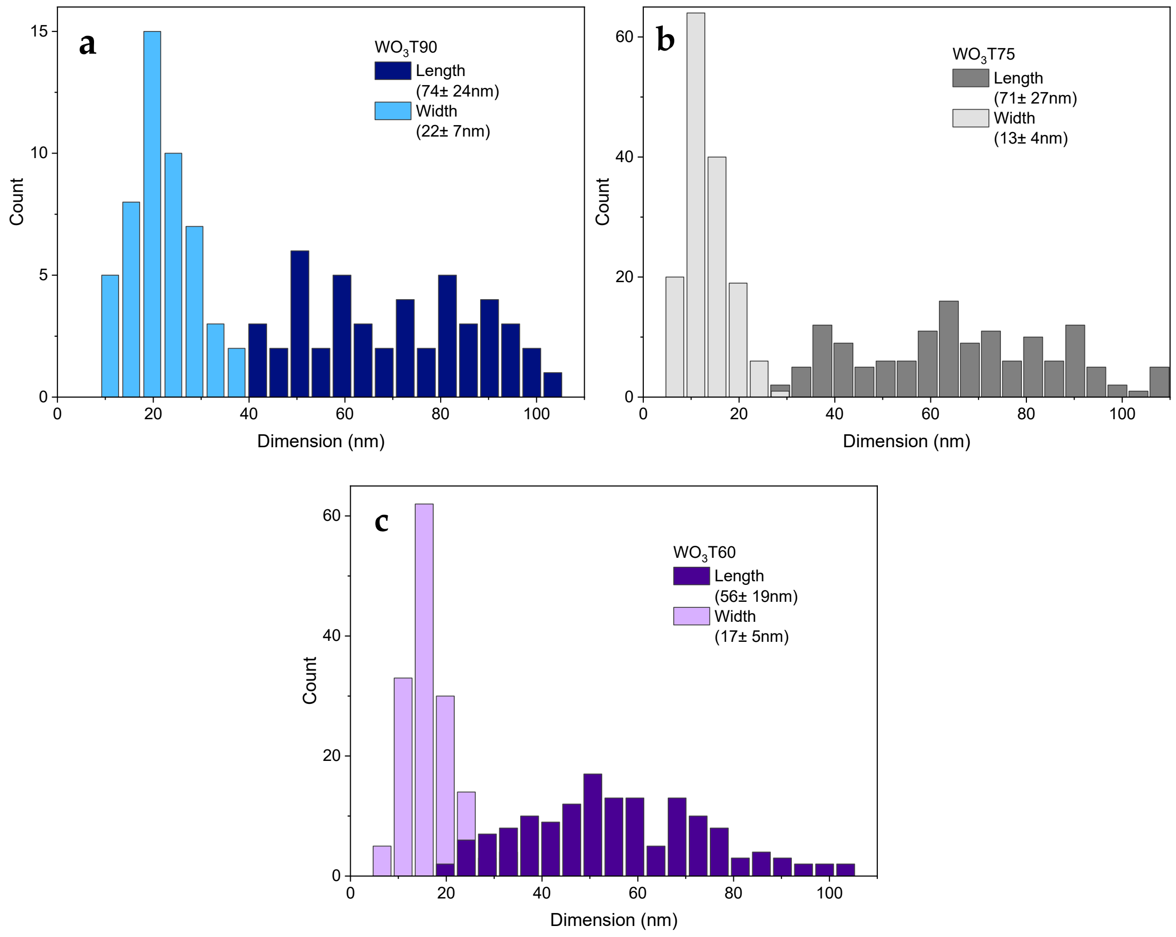

Figure S14. Size distribution analysis of samples (a) WO_3T90, (b) WO_3T75 and (c) WO_3T60.

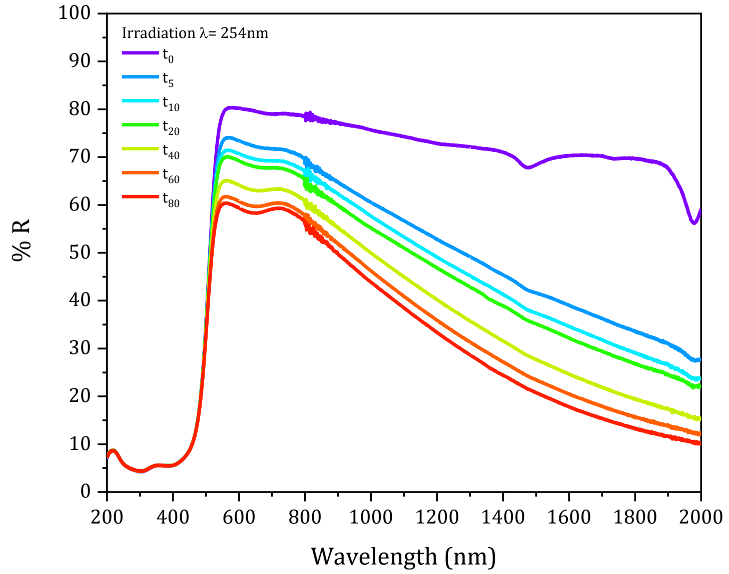

Figure S15. Photochromic kinetic diffuse reflectance spectra of WO_3A1.7, exposed for 80 min at λ = 254 nm

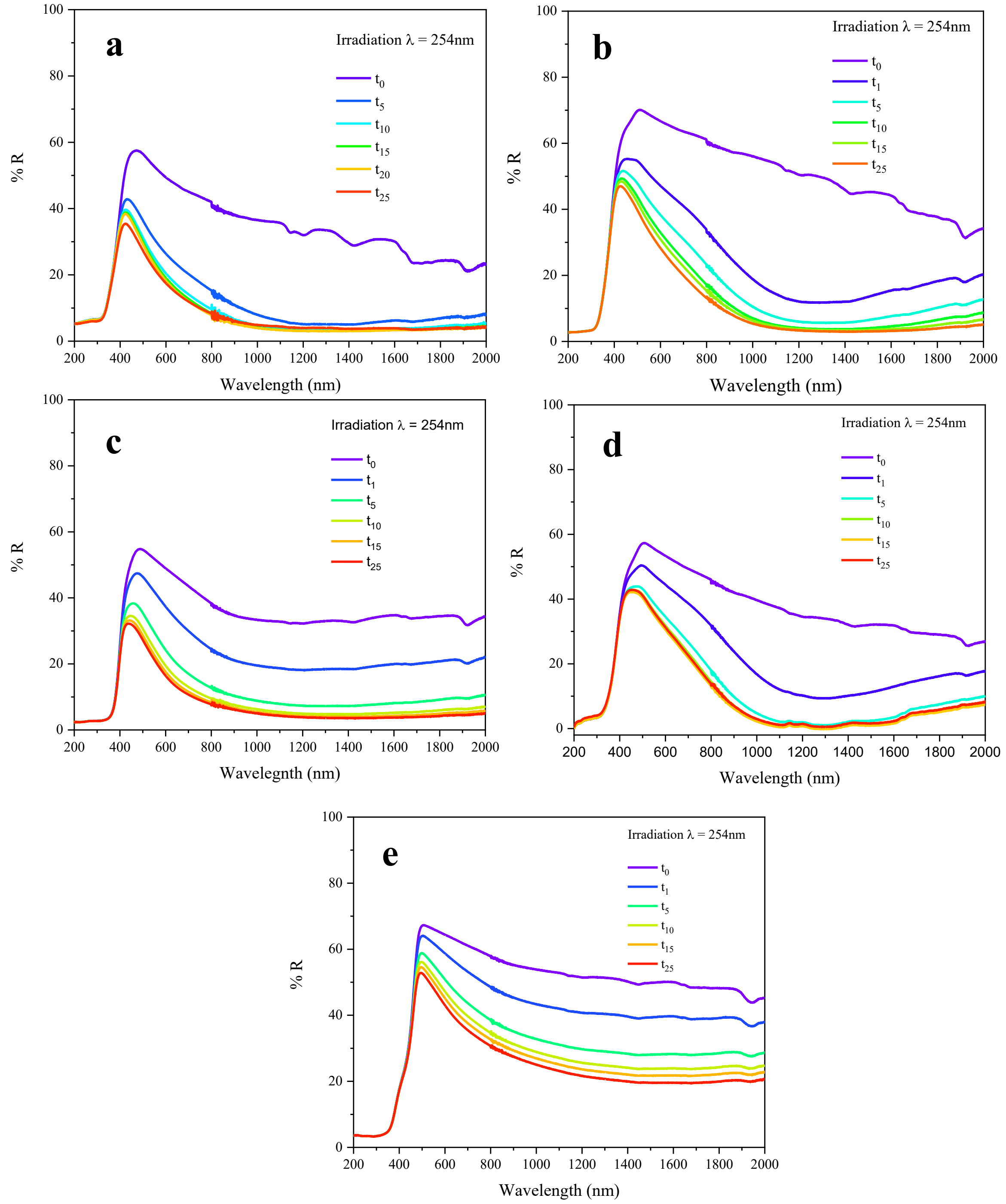

Figure S16. Photochromic kinetic diffuse reflectance spectra of (a) WO_3T90, (b) WO_3T75, (c) WO_3T60, (d) WO_3T45B and (e) WO_3T45Y samples. All the samples have been exposed for 25 min at $\lambda = 254$

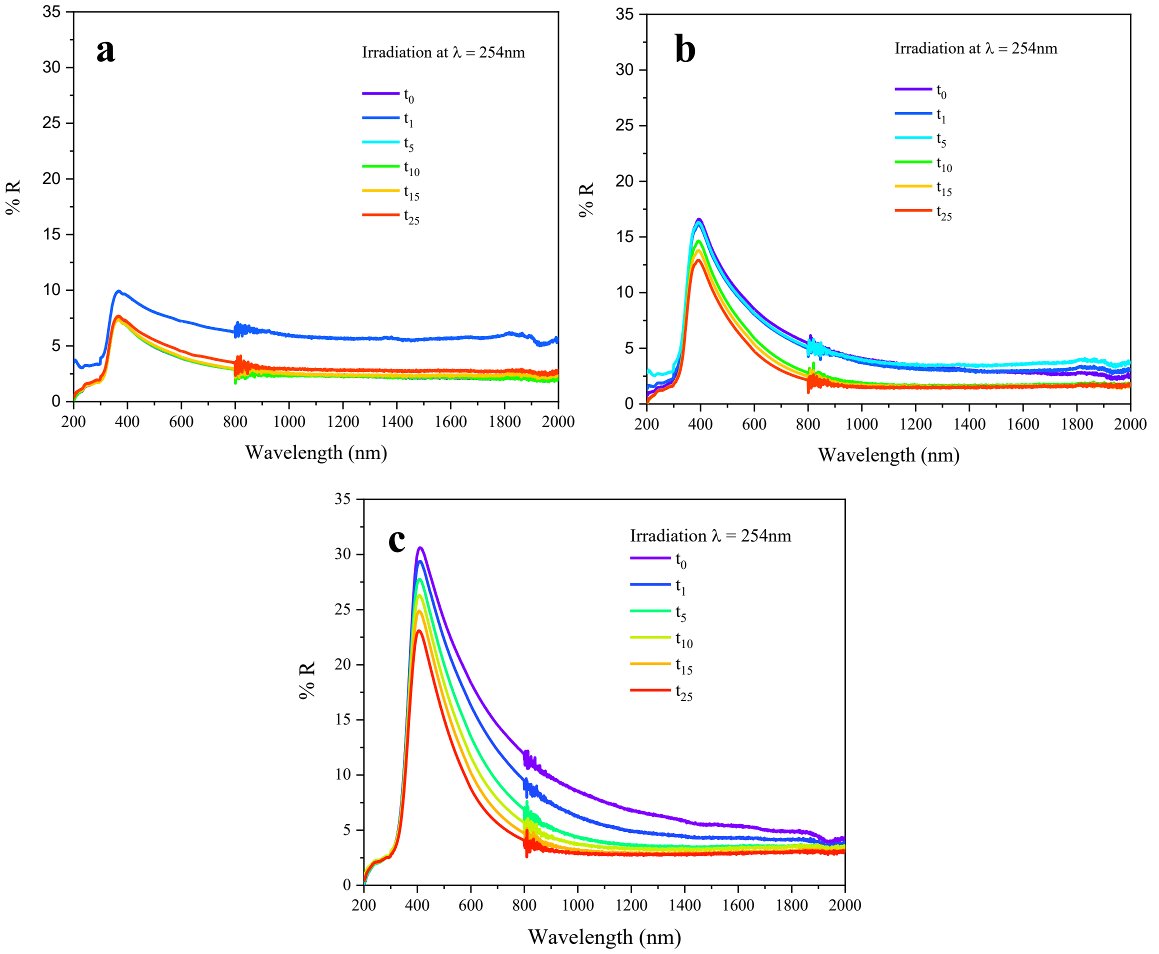

Figure S17. Photochromic kinetic diffuse reflectance spectra of (a) WO_3/PVP-1, (b) WO_3/PVP-5 and (c) WO_3/PVP-10. All the samples have been exposed for 25 min at $\lambda = 254$ nm

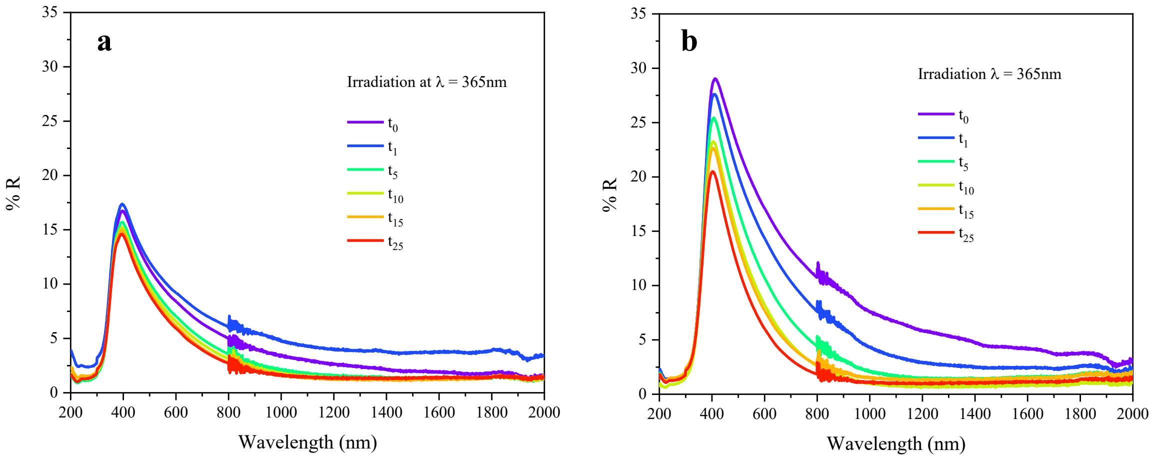

Figure S18. Photochromic kinetic diffuse reflectance spectra of (a) WO_3/PVP-5 and (b) WO_3/PVP-10, exposed for 25 min at $\lambda = 365$ nm

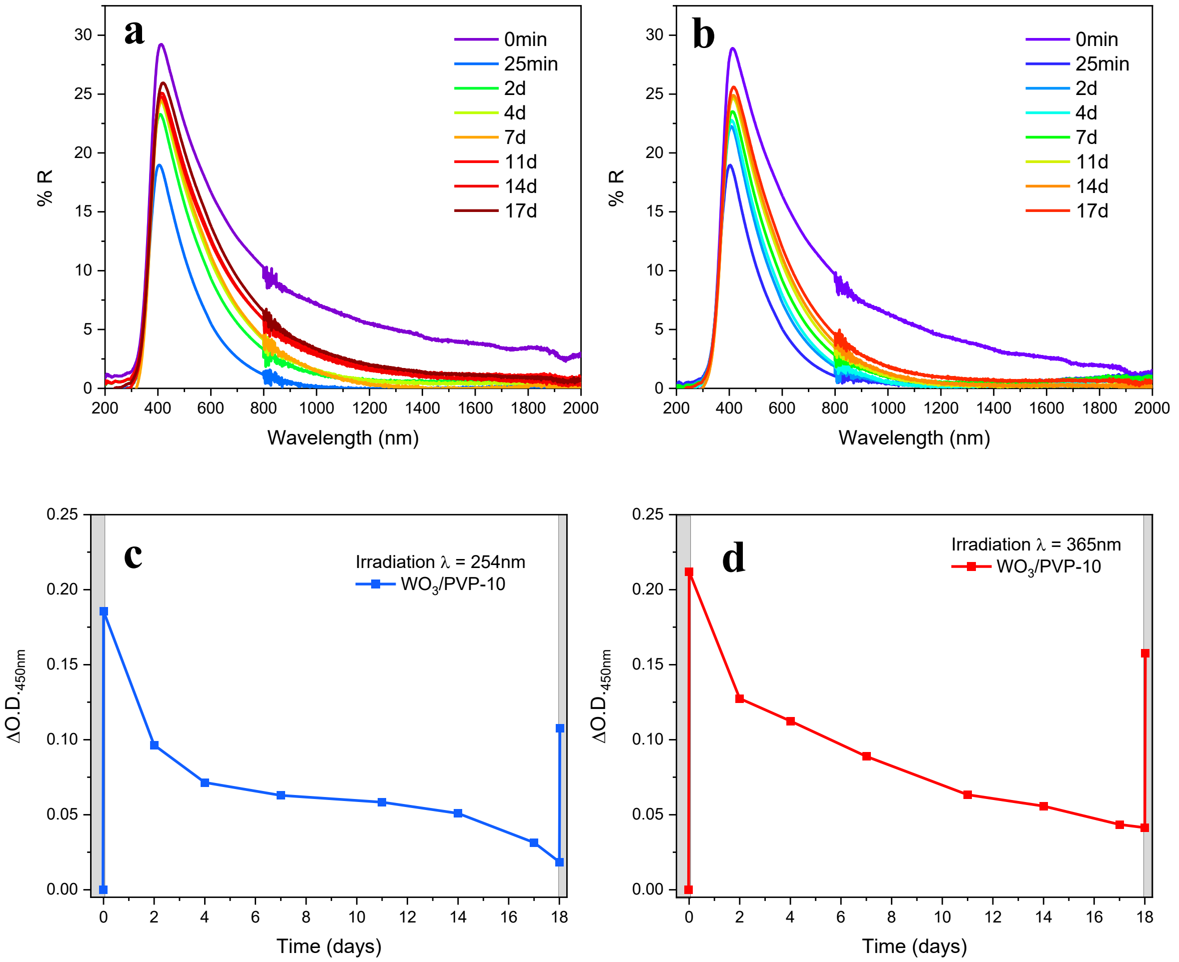

Figure S19. Self-bleaching kinetic of WO_3/PVP-10 sample irradiated at (a) 254nm and (b) 365nm. $\Delta O.D._{450nm}$ bleaching kinetic WO_3/PVP-10 after been irradiated at (c) 254nm and (d) 365nm. The samples after a first irradiation for 25 min have been let to recover in the dark for 18 days before a new irradiation for 25 min.
